# Machine‐Learning‐Aided Advanced Electrochemical Biosensors

**DOI:** 10.1002/adma.202417520

**Published:** 2025-06-09

**Authors:** Andrei Bocan, Roozbeh Siavash Moakhar, Carolina del Real Mata, Max Petkun, Tristan De Iure‐Grimmel, Sripadh Guptha Yedire, Hamed Shieh, Arash Khorrami Jahromi, Sahar Sadat Mahshid, Sara Mahshid

**Affiliations:** ^1^ Department of Bioengineering McGill University Montreal Quebec H3A 0E9 Canada; ^2^ Beeta Biomed Inc. Clinical Innovation Platform Montreal General Hospital Montreal Quebec H3G 1A4 Canada; ^3^ Department of Experimental Medicine McGill University Montreal Quebec H3G 2M1 Canada

**Keywords:** advanced materials, artificial intelligence, biosensors, high‐throughput, nanomaterials, point‐of‐care, wearable

## Abstract

Electrochemical biosensors offer numerous advantages, including high sensitivity, specificity, portability, ease of use, rapid response times, versatility, and multiplexing capability. Advanced materials and nanomaterials enhance electrochemical biosensors by improving sensitivity, response, and portability. Machine learning (ML) integration with electrochemical biosensors is also gaining traction, being particularly promising for addressing challenges such as electrode fouling, interference from non‐target analytes, variability in testing conditions, and inconsistencies across samples. ML enhances data processing and analysis efficiency, generating actionable results with minimal information loss. Additionally, ML is well‐suited for handling large, noisy datasets often generated in continuous monitoring applications. Beyond data analysis, ML can also help optimize biosensor design and function. While extensive research has expanded applications of advanced and nanomaterials‐enhanced electrochemical biosensors and ML in their respective fields, fewer studies explore their combined potential in diagnostics; their synergy holds immense promise for advancing diagnostics and screening. This review highlights recent ML applications in advanced and nanomaterial‐enhanced electrochemical biosensing, categorized into biocatalytic sensing, affinity‐based sensing, bioreceptor‐free sensing, electrochemiluminescence, high‐throughput sensing, and continuous monitoring. Together, these developments underscore the transformative potential of ML‐aided advanced/nanomaterial‐enhanced electrochemical biosensors in diagnostics and screening, paving new pathways in the field.

## Graphical abstract

Electrochemical biosensors are highly sensitive, portable, and versatile. Advanced nanomaterials enhance their performance, while machine learning (ML) improves data analysis, minimizes interference, and optimizes sensor design. Despite progress in both fields, their combined potential in diagnostics remains underexplored. This review highlights ML applications in electrochemical biosensors, showcasing their transformative impact on diagnostics and screening.

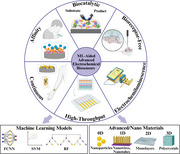

## 1 Introduction

Biosensors are a new generation of sensors that employ a biorecognition element to detect biological responses and a transduction mechanism to convert these responses into measurable signals.^[^ ^1^, ^2^, ^3^, ^4^, ^5^ ^]^ Their application is widespread, ranging from diagnostics and drug screening to food quality control and environmental monitoring.^[^ ^2^, ^3^, ^6^ ^]^ These sensors offer several advantages over traditional analytical methods such as fast running time, low cost, ease‐of‐use, small size, and can be used at the point‐of‐care (POC), while exhibiting good sensitivity and specificity.^[^ ^1^, ^2^, ^3^ ^]^ Biosensors can be employed in conjugation with many different transduction mechanisms to convert a biological signal into a measurable signal. Electrochemical transduction is convenient because the technology is very flexible and amenable to all kinds of different experimental setups, measurements, and analytes.^[^ ^1^, ^2^ ^]^ Electrochemical biosensors are increasingly being integrated with advanced/nanomaterials, such as carbon‐based nanomaterials, metallic nanostructures, and organic‐polymer‐based electronics.^[^ ^7^, ^8^, ^9^, ^10^ ^]^ These materials can be categorized based on their dimensionality: i) 0D materials which have all three dimensions confined to the nanoscale, typically nanoparticles or quantum dots;^[^ ^11^ ^]^ ii) 1D materials which have one dimension significantly larger than the other two, typically nanowires, nanotubes, or fibers; iii) 2D materials which are thin films, monolayers, or sheets, where one dimension is significantly thinner than the other two;^[^ ^11^ ^]^ and iv) 3D materials which have significant volume in all three dimensions and are typically bulk materials.^[^ ^11^ ^]^ Nanomaterials have desirable characteristics, such as a large surface area and excellent electrochemical properties, allowing for improved sensor sensitivity as well as improved overall performance.^[^ ^7^, ^8^, ^12^, ^13^, ^14^, ^15^ ^]^ Organic electronics are gaining interest as they allow for further miniaturization, decreased cost, and more flexible sensors, also driving improvements in electrochemical biosensors.^[^ ^7^, ^16^, ^17^, ^18^, ^19^, ^20^ ^]^

**Figure 1:**
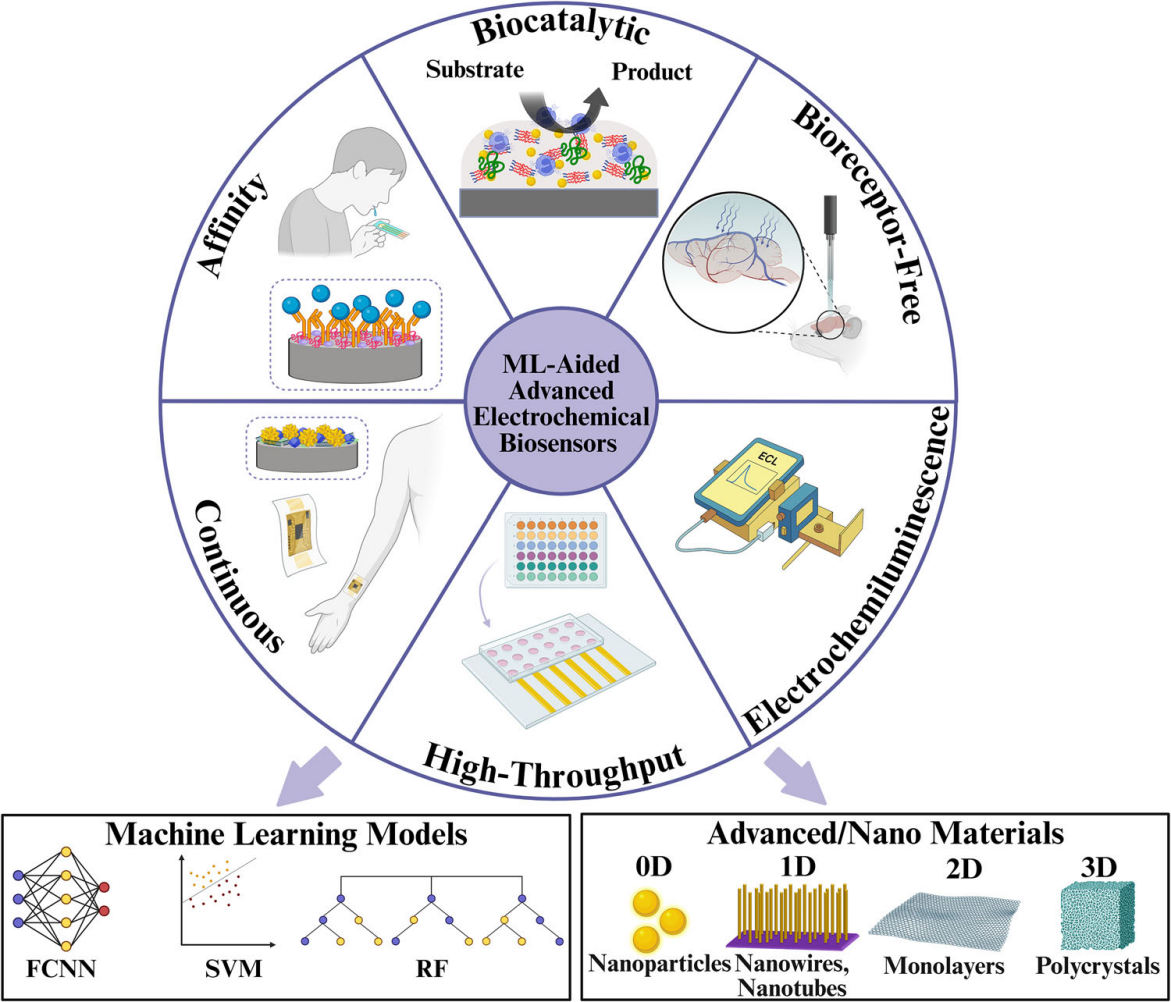
Machine‐learning‐aided advanced electrochemical biosensors. Created in BioRender.

**Figure 2:**
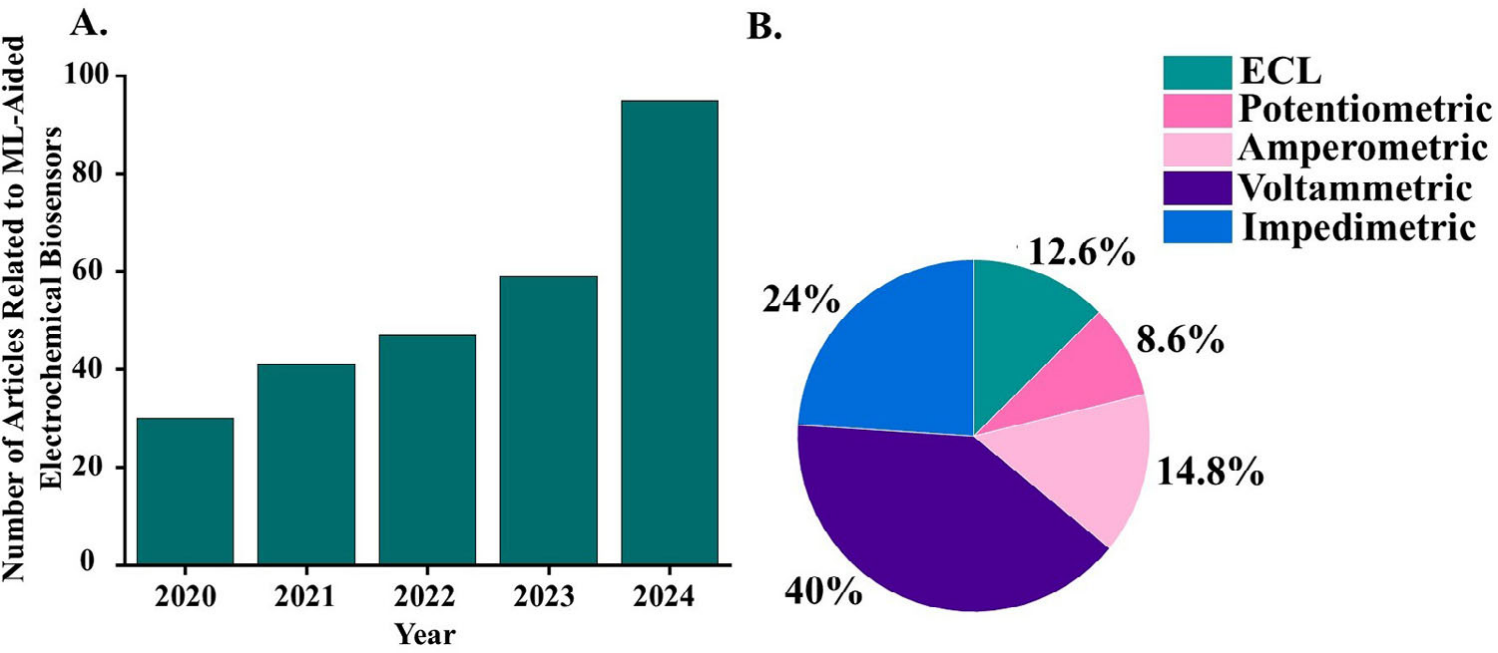
Trends in machine‐learning‐aided advanced electrochemical biosensors. A) Number of articles related to ML‐aided advanced electrochemical biosensors published from 2020–2024 (in the Scopus database). B) Published articles (in Scopus) for ML‐aided advanced electrochemical biosensors, divided by type of electrochemical technique.

However, despite the promise of advanced‐ and nanomaterial‐based sensors, several challenges remain with biosensors at large and electrochemical biosensors specifically, namely poor or inconsistent signal‐to‐noise ratio, instability of biological components, electrode fouling, chemical interferences, and matrix effects.^[^ ^1^, ^2^, ^3^ ^]^ These challenges become more pronounced as electrochemical biosensors are applied in POC settings, where there is less control over sensor operating conditions relative to laboratory settings. This can decrease the signal‐to‐noise ratio, reduce the stability of biological components, and introduce non‐linearities in the signal extracted from the sensor. Additionally, POC applications typically involve the analysis of complex biological samples, where the matrix effect may be more pronounced.^[^ ^3^, ^21^, ^22^, ^23^ ^]^ Successful application of electrochemical biosensors for POC testing will require overcoming these challenges, and while improvements in hardware are key, software advances are likely to be equally important. Machine learning (ML) is an emerging technology with the potential to solve these problems. It has the inherent ability to extract information from large, complex datasets^[^ ^1^, ^2^, ^3^ ^]^ composed of various data modalities, such as images,^[^ ^24^ ^]^ natural language,^[^ ^25^ ^]^ biological sequences,^[^ ^26^ ^]^ and biosensor signals.^[^ ^3^ ^]^ Not only is ML good at recognizing trends and detecting subtleties in complex data, but it is also effective at “unscrambling” data (noise and outlier removal), as well as at isolating the signal of multiple analytes from a single measurement.^[^ ^1^, ^2^, ^3^ ^]^ ML has already been paired with various biosensing modalities. For example, in fluorometric and colorimetric biosensing, ML has been used for more accurate image analysis in sub‐optimal image capture conditions at POC.^[^ ^3^, ^27^ ^]^ For spectroscopic biosensing techniques, such as Raman spectroscopy, and surface‐enhanced Raman spectroscopy (SERS), ML can facilitate the analysis of large spectroscopic data and can help compensate for variability inherent to SERS due to the varying orientation of molecules on the SERS surface.^[^ ^3^, ^28^, ^29^ ^]^ In electrochemical biosensors, ML could help analyze samples with a low signal‐to‐noise ratio and help resolve the analyte of interest from interfering compounds in biological samples.^[^ ^21^ ^]^ Additionally, degradation of biological components, electrode fouling, and variable operating conditions can introduce non‐linearities to the biosensor signal.^[^ ^30^, ^31^, ^32^ ^]^ ML can be highly effective for dealing with these non‐linear relationships within the data. ML can also combine electrochemical data with other data modalities to improve predictive performance^[^ ^33^ ^]^ and can directly provide actionable outputs for the user.^[^ ^34^ ^]^ For these reasons, electrochemical biosensors stand to benefit greatly from ML integration. ML‐aided electrochemical biosensors (**Figure** **1**) have therefore been the subject of increasing interest, as evidenced by recent publishing trends (**Figure** **2**), which show an increase in the number of papers related to ML‐aided electrochemical biosensors published since 2020 (Figure 2A), with ML being applied across various electrochemical sensing modalities (Figure 2B).

In the context of increasing interest in applications of ML for electrochemical biosensors, this review first provides a brief introduction to ML, then examines recent applications of ML across six specific areas of advanced/nanomaterial‐enhanced electrochemical biosensors (henceforth referred to as “advanced electrochemical biosensors”): biocatalytic sensing, affinity‐based sensing, bioreceptor‐free sensing, electrochemiluminescence (ECL), high‐throughput sensing, and continuous monitoring. The first three areas divide electrochemical sensors by the type of (or lack thereof) biorecognition element used. The last three areas explore unique variants of ML‐aided advanced electrochemical biosensors that differ from those explored in the first three sections, with high‐throughput sensing and continuous monitoring being especially relevant in the context of increasing miniaturization and shift to POC applications of electrochemical sensors.^[^ ^22^ ^]^ To the authors’ knowledge, ML applications to these specific areas of advanced electrochemical biosensors have not been reviewed. As each area presents unique challenges, exploring area‐specific ML applications will provide a richer and more specific understanding of how ML can benefit advanced electrochemical biosensors. For a more thorough exploration of ML applications in the domain of advanced electrochemical biosensors, the articles selected for each section include some conventional electrochemical biosensors (i.e., non‐advanced), selected due to the unique ML‐based approaches they propose; these approaches are often also applicable to advanced electrochemical biosensors.

## 2 Machine Learning Overview

We provide a brief overview of machine learning here and refer the reader to previous work for a more thorough review.^[^ ^3^, ^35^, ^36^ ^]^ ML is an umbrella term regrouping a variety of artificial intelligence (AI) algorithms capable of “learning” a task by observing data.^[^ ^1^, ^2^, ^3^, ^6^ ^]^ ML algorithms can learn in a supervised or unsupervised manner. Supervised learning algorithms are trained on “labeled” data with a known output.^[^ ^1^, ^2^, ^3^, ^6^ ^]^ In diagnostics, for instance, the output for a given patient would be whether the patient is positive or negative for a disease. This way, the algorithm learns to recognize trends in the labeled data and eventually makes predictions on the output of new unlabeled data based on these trends.^[^ ^1^, ^2^, ^3^, ^6^ ^]^ Supervised learning is subdivided into classification and regression tasks. When the output variable that must be determined is continuous, like concentration, the task is regression.^[^ ^1^, ^2^, ^3^, ^6^ ^]^ When the output variable is discrete, like assessing whether a patient is positive or not for a disease, the task is classification.^[^ ^1^, ^2^, ^3^, ^6^ ^]^ Unsupervised ML algorithms are not provided with labels. They are either used for clustering, i.e., grouping data based on similarity, or feature extraction and dimensionality reduction, which consists of reducing the number of input features fed to the ML model.^[^ ^1^, ^2^, ^3^, ^6^ ^]^ There is another distinction to be made in ML between unimodal and multimodal ML applications. Unimodal ML evaluates data coming from a single source, while multimodal integrates data from several sources^[^ ^37^ ^]^ (e.g., images and electrochemical signals). Multimodal ML is modeled on clinical decision‐making, where symptoms and test results are weighed against each other to obtain a single, robust outcome.^[^ ^37^ ^]^ In this review, we explore a range of different ML methods, each specific to their given task. Usually, the choice of the right algorithm requires some prior knowledge of the application and the data to be collected. In most cases, model selection and tuning follow a specific workflow.

### 2.1 Machine Learning Workflow

The workflow for ML usually operates as follows. First, raw data must be collected.^[^ ^2^, ^3^ ^]^ This data is often pre‐processed to remove noise, filter‐out outliers, and improve the signal.^[^ ^2^, ^3^ ^]^ Pre‐processing must be done carefully, as it can lead to better results, but can also remove useful information.^[^ ^2^, ^3^ ^]^ With large data (many input variables), it is often desirable to either manually select relevant input variables or apply a dimensionality reduction technique.^[^ ^38^, ^39^ ^]^ Second, an appropriate ML algorithm should be chosen according to the context.^[^ ^2^, ^3^ ^]^ Not all algorithms work the same, they depend on the data collected or the task that is performed. Third, the data must be separated between a training set, a validation set, and a testing set, typically in these proportions: 60% training, 20% validation, and 20% testing.^[^ ^2^, ^3^ ^]^ Training is performed on the training set. This is where the ML algorithm “learns” from the labeled data. Concretely, the model parameter weights are adjusted using a cost function.^[^ ^2^, ^3^ ^]^ Starting from random values, the weights are improved every iteration to minimize the cost.^[^ ^2^, ^3^ ^]^ Validation is performed after training using new unseen data from the validation set. During this operation, the model hyperparameters are tuned. The hyperparameters are responsible for the overall architecture of the model.^[^ ^2^, ^3^ ^]^ Their values are varied to improve the prediction accuracy of the model on the validation set.^[^ ^2^, ^3^ ^]^ The testing step is performed on the test set, consisting of unseen data. The accuracy of the model is calculated by comparing its predictions of the output to the true output values from the test set.^[^ ^2^, ^3^ ^]^ Finally, if several models are evaluated, the one with the best performance is selected.^[^ ^2^, ^3^ ^]^ Sometimes, cross‐validation is performed over the whole dataset rather than using the 60/20/20 split. Cross‐validation is viewed as a robust measurement for out‐of‐sample accuracy when there is little data available.^[^ ^40^ ^]^ When performing cross‐validation, the data is divided into subsets of equal size.^[^ ^41^ ^]^ The model is repeatedly trained on all but one of the subsets and then tested on the remaining one for accuracy. This operation is repeated as many times as there are subsets, each time excluding a new subset and using it as the test set. Taking the average of the performance metric values gives a more accurate measure of the performance of the model. Although the ML workflow may vary from one application to another, these are the general steps. As mentioned above, training, hyperparameter tuning (validation), and testing all require knowledge of relevant performance metrics. Also, metrics are crucial for comparing to other models in terms of performance analysis. Evaluation metrics for classification and regression ML algorithms have already been discussed extensively in other papers. We kindly recommend the work by Hoffman et al.^[^ ^42^ ^]^ as reference. Metrics for classification tasks generally include accuracy, precision, sensitivity, receiver operating characteristic (ROC) curve, area under the receiver operating characteristic curve (AUROC), recall, and F1 score. For regression, the coefficient of determination (*R* ^2^), mean absolute error (MAE), mean square error (MSE), and root mean square error (RMSE) are typically used. An important consideration when evaluating ML model performance is generalizability: ML models can often fail to generalize, performing well on the training data but poorly on unseen test data; this may be due to overfitting, where the model is not capturing the true relationships between input and target variables, and is instead learning spurious correlations present only in the training data. It may also be that the test data is simply too different from the training data. These issues can be addressed by careful feature and model selection, by ensuring that no data leakage occurs (i.e., where the model “sees” some of the supposedly unseen data in training), and by careful selection of the unseen test data.^[^ ^35^, ^43^, ^44^ ^]^

### 2.2 Machine Learning Algorithms

The workflow detailed above remains similar regardless of the model used. Here, we provide a brief overview of the most common ML algorithms used with advanced electrochemical biosensors. These can be divided into so‐called “shallow” ML algorithms and deep learning algorithms. Shallow ML algorithms include support vector machines (SVM), decision trees (DT), random forest (RF), gradient‐boosted trees (GBT), and shallow artificial neural networks (ANN).^[^ ^45^, ^46^ ^]^ Deep learning algorithms encompass neural networks with three or more (usually significantly more) layers.^[^ ^45^, ^46^, ^47^ ^]^ Shallow ML models are generally smaller (i.e., fewer parameters), their performance does not scale with the training dataset size, they are less expressive (i.e., less capable of modeling complex non‐linearities), and they are usually trained on features extracted from raw data. However, these models require fewer training data to achieve acceptable performance.^[^ ^46^ ^]^ Conversely, deep learning models are generally larger (i.e., more parameters) and more expressive, with performance scaling with training dataset size, but they require more training data to perform acceptably. They are also capable of handling raw data directly, effectively extracting their own features from it.^[^ ^46^ ^]^ There is no objectively superior ML model: different models will be suitable for different applications; the choice of model will depend on the task and data at hand. Here, we first survey shallow ML models, followed by a brief discussion of neural networks. Among the most popular traditional ML models are SVMs. SVMs are supervised ML algorithms that were originally designed for binary, linear classification.^[^ ^48^ ^]^ They learn by finding the largest gap (called margin) that exists between the furthermost instances of each class (called support vectors).^[^ ^48^ ^]^ SVM applications nowadays have been extended to multi‐class problems, and non‐linear problems using kernels.^[^ ^48^ ^]^ Kernels transform the data from the input space to a higher dimensional space that is easily separable with a linear surface (called hyperplane).^[^ ^48^ ^]^ SVMs work well when there are clear margins between classes.^[^ ^3^ ^]^ They are useful for analyzing overlapping signals and when the sample number is small.^[^ ^3^ ^]^ They do not perform well with large data, when there are several classes, and when data is noisy.^[^ ^3^ ^]^

RF is a supervised, ensemble method that performs majority voting with many DT.^[^ ^3^ ^]^ DT are hierarchical structures made of nodes, branches, and leaves.^[^ ^3^, ^49^ ^]^ Every decision taken by the model towards a prediction is in the form of a node.^[^ ^3^, ^49^ ^]^ Branches leaving a node represent values that the node can assume.^[^ ^3^, ^49^ ^]^ The endpoints, called leaves, represent probability density class distribution for classification or value distribution for regression.^[^ ^3^, ^49^ ^]^ Because of majority voting, RF is better at handling noise and overcoming overfitting than a single DT. However, as the number of trees increases in RF, decision‐making becomes slower, hindering its real‐time decision‐making abilities.^[^ ^3^ ^]^ GBT, which includes eXtreme Gradient Boosting (XGBoost), is an ensemble method in which DT are added sequentially (as opposed to in parallel in RF) to correct for the loss of previous models until no noticeable improvement can be detected.^[^ ^50^, ^51^ ^]^ Loss is calculated as the difference between the predicted and true labels. Gradient descent is used to minimize the loss when adding new models. Regularization is also applied to avoid overfitting.^[^ ^51^ ^]^ GBT can be applied to classification and regression.^[^ ^51^ ^]^ XGBoost in particular is state‐of‐the‐art with high computational speed and model performance.^[^ ^50^ ^]^

K‐nearest neighbors (KNN) is a supervised ML algorithm used for classification.^[^ ^52^ ^]^ When evaluating a test data point, the algorithm sorts through the entire training set to find the k‐most similar training examples in the feature space.^[^ ^52^ ^]^ A majority voting is performed between the k neighbors, and the dominant class is selected as the prediction for the test point.^[^ ^52^ ^]^ This is a very simple algorithm, easy to implement and interpret.^[^ ^3^ ^]^ Model training only requires storing the training data in a memory location. However, the model does not translate well to high‐dimensional problems.^[^ ^3^ ^]^ Also, determining the value of k requires tuning as it is problem‐specific.

ANN refers to a class of ML models that are inspired by biological neural networks.^[^ ^3^, ^53^ ^]^ The nodes in ANNs are connected and work together much like neurons.^[^ ^3^, ^53^ ^]^ The structure of ANNs consists of an input layer, an output layer, and a number of hidden layers in between, with a number of nodes found in each layer. ANNs are feedforward, meaning that the information proceeds from one layer to the next in a unidirectional fashion.^[^ ^53^ ^]^ For simple ANNs (i.e., shallow ML), the number of hidden layers tends to remain in the 0–3 range. Deep neural networks (DNNs) will have tens to hundreds of hidden layers.^[^ ^46^, ^47^ ^]^ ANNs are also known as fully connected neural networks (FCNNs), whereby each node in one layer is linked to all nodes in the preceding layer. Conversely, in a convolutional neural network (CNN), each node in a layer is linked to a limited set of nodes in the preceding layer. CNNs consider the spatial resolution and are therefore especially well‐suited to image analysis tasks. Instead of feeding individual pixels to the nodes, whole patches of pixels are analyzed together to preserve the spatial relation between them. Features are extracted using convolutional filters to produce feature maps, which are then passed on as input to the following layers. The final feature maps are compressed and typically fed to an ANN.^[^ ^54^, ^55^ ^]^ Recurrent neural networks (RNNs) are another variant where the output from a previous step can circulate back in the form of a feedback loop, thus keeping a record of past information, RNNs are therefore well‐suited to the analysis of time series.

Principal component analysis (PCA) is not a ML algorithm per se, but rather a multivariate statistical technique for dimensionality reduction that is extensively used in several ML applications.^[^ ^56^ ^]^ In PCA, new variables called “principal components” (PCs) are all orthogonal linear combinations of the original variables. These PCs capture the greatest axes of variation within a given dataset, with the first PC carrying the largest variance, followed by the subsequent components in decreasing order. This allows for reducing the number of variables within a data set. A dataset can have too many variables relative to the number of samples (i.e., “wide” data), which can lead to overfitting. PCA can help reduce this by effectively grouping the relevant information found in a large number of variables into a smaller number of explanatory PCs.^[^ ^44^ ^]^

Overall, this manuscript reviews a broad range of ML models applied in electrochemical biosensing, including those discussed above and others: (i)Tree‐based methods:decision trees, random forest, XGBoost, ensemble bagged trees, AdaBoost; (ii) Neural networks: DNN, CNN, RNN, radial basis function (RBF) neural networks, convolutional autoencoder; (iii) Regression & Linear Methods: ridge regression, linear maximum likelihood estimation, Gaussian process regression, instance‐based learning such as KNN, support vector machine/regression, Sure Independence Screening (SISSO); (iv) Dimensionality reduction & feature selection: PCA, linear discriminant analysis; (v) Optimization & search methods: Particle swarm oOptimization. The following section will briefly detail how various ML algorithms can be applied to help solve key issues in electrochemical biosensing.

### 2.3 Machine Learning for Electrochemical Biosensing

The algorithms detailed above all have different characteristics and may be suitable for different applications. Generally, within electrochemical biosensing, ML algorithms could help with reducing the effect of electrode fouling, variability, noise, and matrix effects in electrochemical measurements, improving sensitivity and specificity. Here, we will examine these various challenges, and how different ML algorithms may help in overcoming them.

#### 2.3.1 Electrode Fouling

Fouling typically introduces non‐linearities which can be accounted for using non‐linear ML models.^[^ ^1^, ^2^ ^]^ In a study by Aiassa et al. in 2021, SVM was applied with a RBF kernel to compensate for electro‐polymerization occurring at the electrode surface.^[^ ^30^ ^]^ Propofol detection in this application was increased from 68.9% and 33.3% in PBS and human serum respectively, to 98.9% and 100%.^[^ ^30^ ^]^ Similarly, an ANN model was developed by De Jaegher et al.^[^ ^57^ ^]^ to describe the fouling of ion‐exchange membranes in electrodialysis. ANN modeling was useful in capturing the non‐linear behavior of particle attachment, thus improving the predictive power of the model.

#### 2.3.2 Variability and Generalization

Leveraging large, diversified training datasets enhances the generalization ability of ML models. As highlighted by Giordano et al., expanding training datasets with varied experimental conditions allows models to better adapt to unforeseen variability, ultimately improving prediction performance and reproducibility across different electrochemical systems.^[^ ^3^ ^]^ As well, probing the experimental conditions during data collection allows ML models to account for variability when making their predictions. A low‐concentration, electrochemical glucose detection system published in 2024 tested several ML models including DT, ANN, and RF, and determined that XGBoost was the most accurate and robust over a variety of experimental conditions.^[^ ^58^ ^]^ Typically, ensemble methods are recognized as powerful tools for managing variability.^[^ ^59^ ^]^

#### 2.3.3 Noise and Matrix Effect

When tackling applications with noise or interference from the matrix effect, dimensionality reduction techniques such as PCA help identify the features with the greatest variation, removing any interfering or baseline signals.^[^ ^59^ ^]^ The ANN family of models also exhibits strong feature‐extraction and pattern recognition abilities, making them robust to noise and variability.^[^ ^59^ ^]^ CNNs and RNNs can differentiate minute electrochemical signal variations indicative of low analyte concentration from noise thanks to their ability to deal with complex, nonlinear patterns within high dimensional data. SVMs, which use kernels to transform linearly inseparable data into higher dimensions, facilitate matrix unscrambling and improve selectivity in the presence of similar analytes or interfering analytes.^[^ ^59^ ^]^

By combining these strategies, ML algorithms offer a comprehensive approach to mitigating challenges inherent in electrochemical applications, paving the way for more reliable, robust, and sensitive detection systems, the following sections will provide detailed examples illustrating this.

## 3 Machine‐Learning‐Aided Advanced Electrochemical Biosensors

### 3.1 Biocatalytic‐Based Biosensors

In 1962, Clark and Lyons proposed the first electrochemical biosensor, making use of electrode‐immobilized glucose oxidase (GOx) enzyme for detection of glucose in blood;^[^ ^60^ ^]^ sensors such as this, making use of enzymes as bio‐recognition elements, are known as “biocatalytic”, or enzymatic, electrochemical biosensors, and are still relevant today.^[^ ^61^, ^62^ ^]^ In these sensors, the enzyme recognizes the analyte of interest and catalyzes a reaction resulting in measurable changes in current or voltage at the electrode surface.^[^ ^22^, ^62^, ^63^ ^]^ Different enzymatic electrochemical biosensing modalities exist: first‐generation biocatalytic sensors rely on oxygen as a natural mediator to shuttle electrons between the enzyme redox active center and the electrode surface, while more recent biocatalytic sensors either utilize artificial redox mediators, or direct electron transfer from the enzyme to the electrode.^[^ ^64^, ^65^, ^66^, ^67^ ^]^ Electrons transferred result in a measurable signal at the electrode, typically detected using amperometry or potentiometry, which can be correlated to the analyte concentration.^[^ ^18^, ^66^ ^]^ The use of enzymes as bio‐recognition elements allows for rapid reaction times and high specificity; conversely, enzymes are subject to a gradual loss in activity over time and are sensitive to changes in operating conditions, negatively impacting the reliability of biocatalytic sensor readings.^[^ ^22^, ^62^, ^68^, ^69^ ^]^ ML, through multi‐modal learning, can overcome these pitfalls by combining electrochemical sensor data with sensor operating condition data (e.g., ambient temperature, sample pH) or with other data modalities (e.g., spectroscopic data). ML has therefore been combined with biocatalytic sensors in various use cases.

**Figure 3:**
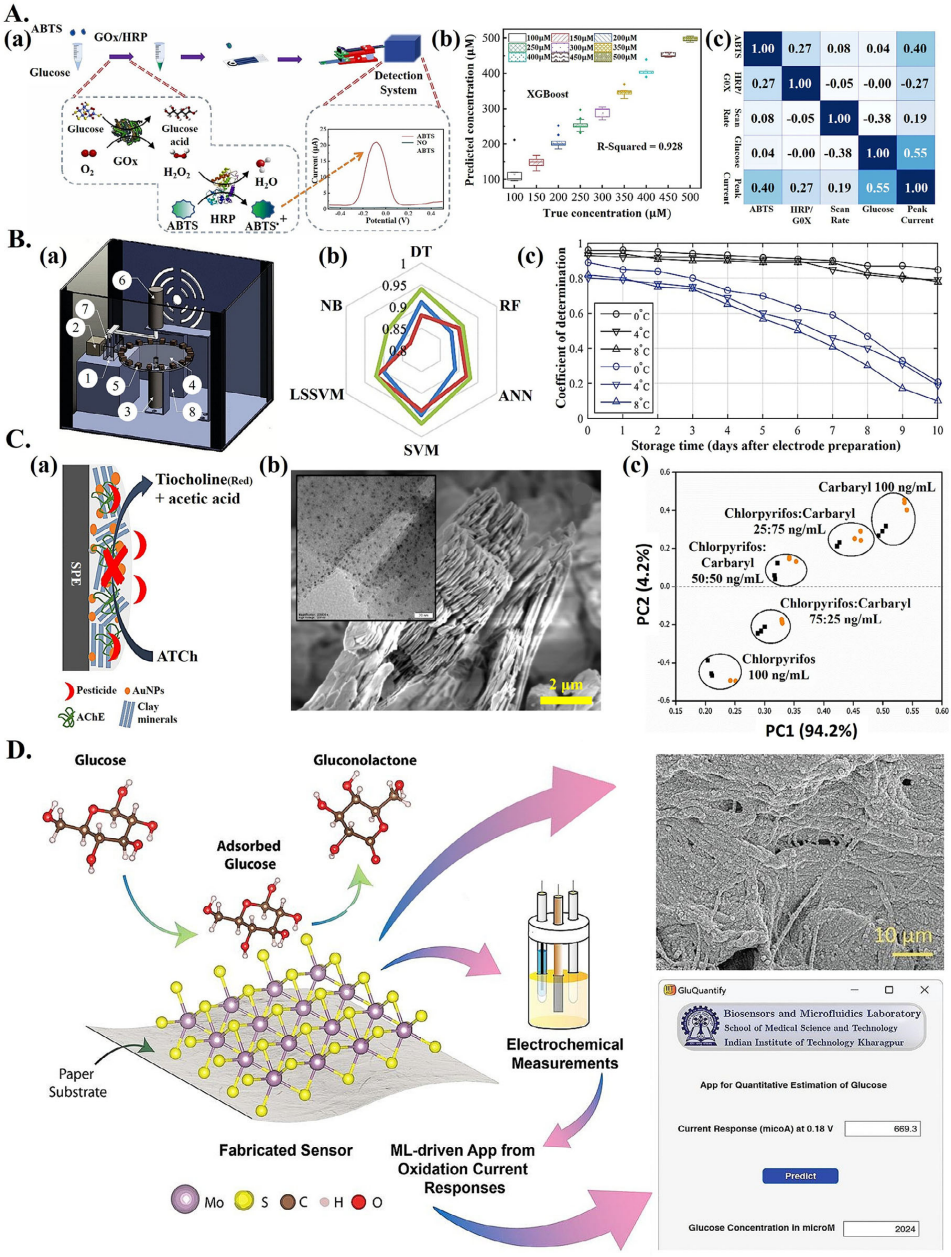
ML‐aided advanced biocatalytic electrochemical biosensors. A) ML for DPV‐based glucose detection. a) GOx produces hydrogen peroxide (H_2_O_2_), HRP oxidizes ABTS in the presence of H_2_O_2,_ causing a detectable DPV response when ABTS is present. b) Best model (XGBoost) performance for glucose detection at various concentrations. c) Correlation heatmap for experimental variables. a–c) Reproduced with permission.^[^ ^58^ ^]^ Copyright 2024, Elsevier. B) ML for combined electrochemical and spectroscopic nitrate biosensing. a) 3D rendering of device, showing quartz cuvette (1), photodiode (2), step motors (3,6), circular frame (4) with UV–vis–NIR LEDs (5), argon purge line (7), and container for electrode storage (8). b) Spider‐graph for *R* ^2^ achieved by various ML models tested for nitrate quantification; colored lines correspond to different redox mediators tested. c) Performance (*R* ^2^) over time for SVM method with (black line) and without (blue line) considering operating and sample conditions; legend showing different storage temperatures. a–c) Reproduced with permission.^[^ ^70^ ^]^ Copyright 2018, IEEE. C) PCA‐aided advanced biocatalytic sensor for pesticide detection from amperometric data. a) AChE immobilized on electrode modified with AuNPs and clay minerals, for pesticide detection. b) SEM image of clay mineral with TEM image of AuNP‐decorated clay mineral in inset. c) Inhibition data from different sensors is analyzed with PCA, mixtures of Chlorpyrifos and Carbaryl in different ratios can be separated in synthetic (black dots) and real (orange dots) samples. a–c) Reproduced with permission.^[^ ^77^ ^]^ Copyright 2022, American Chemical Society. D) ML‐aided non‐enzymatic advanced paper‐based biocatalytic glucose sensor modified with MoS_2_. FESEM image of MoS_2_‐modified paper substrate shown (top right), along with GluQuantify app interface (bottom right). Reproduced with permission.^[^ ^78^ ^]^ Copyright 2024, Elsevier.

The first notable use case is for managing variability in sensor operating conditions. For example, Zhang et al.^[^ ^58^ ^]^ proposed a ML‐aided differential pulse voltammetry (DPV) biocatalytic sensor for more reliable glucose detection in varying operating conditions. GOx and horseradish peroxidase (HRP) were used for glucose detection; ABTS^+^, a result of ABTS oxidation catalyzed by HRP, produced a measurable DPV signal, with the peak current correlated to glucose concentration (**Figure** **3**A,a). Various operating factors (e.g., amount of ABTS, scan rate) were also found to be correlated with the peak current (Figure 3A,c). With optimal operating conditions, the peak current and glucose concentration were linearly correlated, in sub‐optimal conditions this relationship became non‐linear. An ML‐based approach was proposed to remedy this. An XGBoost model was trained with electrochemical data (peak current) combined with operating factor data and achieved a test set *R* ^2^ of 0.928 for glucose concentration prediction (Figure 3A,b, **Table** **1**). In a similar work, Vakilian and Massah^[^ ^70^ ^]^ combined cyclic voltammetry (CV) with ultraviolet‐visible‐near‐infrared (UV–vis–NIR) spectroscopy and used multi‐modal learning for analysis to enable accurate nitrate‐reductase‐based nitrate detection in varying operating conditions. CV data, spectroscopic data (150–1000 nm range absorption), operating conditions (electrode storage time and working electrode temperature), and sample characteristics (sample type and pH) were fed to various ML models for analysis. The electrochemical and spectroscopic detection hardware were integrated into a single portable device (Figure 3B,a). Different ML models were trained and tested with data from various liquid samples (water, fruit juice, plant extract, and human saliva). The best performer was SVM, with an *R* ^2^ of up to 0.97 for nitrate concentration prediction (Figure 3B,b and Table 1). Testing of the device over ten days showed that accuracy decreased significantly when operating conditions and sample characteristics were not provided as inputs to the ML model (Figure 3B,c), a similar decrease was observed when spectroscopic features were withheld. Providing all features to the ML model allowed the *R* ^2^ to be maintained above 0.85 after ten days. Thus, as biocatalytic electrochemical biosensors shift from controlled laboratory settings to POC settings, where operating conditions are more variable,^[^ ^22^, ^71^ ^]^ ML can help maintain sensor reliability and accuracy.

**Table 1:** Machine‐learning‐aided biocatalytic electrochemical biosensors.

Target	Materials	Fluid^a)^	EC method	ML type	Metrics	Year/Refs.
Glucose	Carbon WE	PBS	DPV	XGBoost	*R* ^2^ = 0.928; RMSE = 31.034 µmol L^−1^	2024^[^ ^58^ ^]^
Nitrate	Glassy carbon disk WE	Water, Fruit juice, Plant extract, Human saliva	CV	SVM	*R* ^2^ = 0.97	2018^[^ ^70^ ^]^
Nitrate	Glassy carbon disk WE	Nitrate standard	CV	SVM	*R* ^2^ = 0.93; MSE = 0.0016 µm^2^	2019^[^ ^32^ ^]^
Glucose sensor sensitivity	Gold‐coated medical grade lancets, stabilized with PCL embedded in PDMS	Water/Artificial ISF	Amperometry, EIS	RF	MAE = 1.50 nA mm ^−1^	2023^[^ ^73^ ^]^
RBC Count	EPPGE modified with MWCNTs‐IL WE	Blood (PBS‐diluted RBC solutions)	Amperometry	RBF – FCNN	RMSE = 0.05 × 10^12^ RBCs L^−1^	2024^[^ ^76^ ^]^
Chlorpyrifos Carbaryl	Screen‐printed carbon WE, modified with clay minerals / AuNPs	Pesticide solution Carrot Juice	Amperometry	PCA	LOD (real sample) = 0.5 ng mL^−1^	2022^[^ ^77^ ^]^
Glucose	MoS_2_ on chromatography paper as WE	Serum	DPV	SVR	Acc. (% recovery) = 99.64 ± 1.60%	2024^[^ ^78^ ^]^

^a)^ Biological fluids are of human origin unless otherwise specified.

Aside from variable operating conditions, a decrease in enzyme activity over time is another concern with biocatalytic sensors. By integrating the “age” of the immobilized enzyme (date and time of enzyme immobilization) into the predictive model, reliable sensor performance can be maintained even as enzyme activity decreases.^[^ ^32^ ^]^ A multi‐modal SVM model, integrating CV data as well as the age of the immobilized nitrate reductase enzyme, was trained for nitrate detection. Data for training and testing were collected from nitrate standard solutions. The model achieved an *R* ^2^ of 0.93 for nitrate concentration prediction (Table 1), with slight decreases in performance when tested in the presence of various interferants (e.g., nitrite, fluoride, chloride). As the enzyme “aged”, performance decreased reaching *R* ^2^ values close to 0.8 after 10 days, at which point the enzyme required replacement. The device was also tested with samples of unknown nitrate concentration of various origins (e.g., lake water, cucumber juice, orange juice), and performed well in comparison with a state‐of‐the‐art spectroscopic method. Model performance over 10 days using only electrochemical features was not evaluated; this could have provided a performance baseline when enzyme age was not considered. Still, multi‐modal learning showed promise for overcoming the loss in enzyme activity. Along with decreasing enzyme activity, biofouling of the electrode surface is also a concern, especially in continuous glucose monitors (CGM), which are enzymatic electrochemical sensors implanted in vivo, under the skin.^[^ ^72^ ^]^ Sharma et al.^[^ ^73^ ^]^ used ML to predict the sensitivity of a CGM‐like glucose sensor in the presence of biofouling. A RF model was trained to predict the sensor sensitivity when provided with electrochemical impedance spectroscopy (EIS) parameters (double‐layer capacitance and charge‐transfer resistance), amperometry was used to measure the sensor sensitivity to obtain a ground truth. Sensor needles consisting of medical‐grade lancets, coated with gold and stabilized with the help of hard polymer (polycaprolactone, PCL) embedded with soft polymer (Polydimethylsiloxane, PDMS) were used. The needles were functionalized with GOx enzyme for glucose detection. Amperometry and EIS readings were taken, every two days for three weeks, with the sensor needles in aqueous glucose solutions; the needles were stored in artificial interstitial fluid in‐between readings. Using the RF regression model, a MAE of 1.50 nA mm ^−1^ for the predicted sensitivity was achieved (Table 1), whereas sensitivity values were on the scale of 7–11 nA mm ^−1^. For future work, amperometric readings could be combined with EIS parameters and fed to a ML model for a glucose concentration prediction task. In CGMs, biofouling and enzyme degradation are significant issues that require frequent sensor calibration and electrode replacement,^[^ ^74^, ^75^ ^]^ placing an additional burden on the user; ML has shown potential for dealing with these issues, and a successful ML‐CGM pairing could prove beneficial to CGM users.

Apart from accounting for variations in operating conditions, loss of enzyme activity, and biofouling, ML has been coupled with biocatalytic advanced electrochemical sensors in a few other interesting ways. For instance, Jalili and Jalalvand^[^ ^76^ ^]^ proposed to predict red blood cell (RBC) count from electrochemical data, using ML. Detection was based on the reduction of blood oxygen by the enzyme catalase. The proposed sensor was based on an edge plane pyrolytic graphite electrode (EPPGE) modified with a thin layer of multiwalled carbon nanotubes‐ionic liquid (MWCNTs‐IL). Modification with MWCNTs‐IL resulted in a composite film on the surface of the EPPGE, this film combined the advantages of CNTs and IL, improving the sensor sensitivity. The sensor amperometric response was found to be linearly correlated with the number of RBCs. A 3‐layer RBF‐FCNN was trained on amperometric data collected from calibration solutions with known amounts of RBCs. This model achieved an RMSE of 0.05 (× 10^12^/*L*) on the validation set (Table 1), while actual concentration values ranged from 0.25–5  × 10^12^ L^−1^. In testing with samples from human subjects, the RBC count predicted by the biosensor was close to the values determined by a hemocytometer (maximal difference of 0.01 × 10^12^ L^−1^). In another work, PCA was used to analyze amperometric data from four sensors, each using a different clay mineral/gold nanoparticle (AuNP) nanocomposite as a base material for immobilization of the enzyme acetylcholinesterase (AChE) (Figure 3C,a,b), with the aim of detecting the pesticides Chlorpyrifos and Carbaryl,^[^ ^77^ ^]^ which are harmful to human health. The use of different clay minerals resulted in varied electrochemical responses, allowing the pesticides to be distinguished from each other without the use of multiple enzymes. Amperometric data were collected with the four sensors, with both synthetic and real (carrot juice) pesticide samples. Using PCA, it was possible to differentiate between Chlorpyrifos, Carbaryl, and a mixture of the two, as well as their different concentrations; it was also possible to differentiate between Chlorpyrifos and Carbaryl mixtures in different ratios (Figure 3C,c). This study explored ML to a limited extent. Since PCA visualization showed the possibility of distinguishing between the different pesticides and their concentrations, the next step would be to train classifier (for pesticide identification) and regressor (to determine concentration) models on the PCA‐transformed data. While ML‐aided biocatalytic sensors have shown promise in glucose and nitrate detection, these studies suggest that they have broader applicability, with the possibility of additional uses emerging in the future.

Though ML has helped overcome issues inherent to enzyme‐based biocatalytic electrochemical biosensors, interest in non‐enzymatic biocatalytic biosensors has been increasing.^[^ ^78^, ^79^ ^]^ These sensors are not faced with the same stability issues and susceptibility to environmental conditions as their enzymatic counterparts. However, they face challenges when it comes to specificity to the analyte‐of‐interest.^[^ ^78^, ^79^ ^]^ To overcome issues with low specificity, Pal et al.^[^ ^78^ ^]^ proposed an electrochemical glucose sensor based on molybdenum disulfide (MoS_2_), MoS_2_ was hydrothermally grown over chromatography paper substrate and showed excellent biocatalytic properties following exposure to glucose solution. MoS_2_ microflower structures formed within the paper substrate cellulose fiber network augmented the electrode surface area and facilitated increased interaction with the analyte. DPV was used to measure the electrochemical signal from glucose‐spiked human serum samples. Peak oxidation currents from DPV measurements were analyzed by a support vector regression (SVR) model, which was integrated into a standalone desktop application known as “GluQuantify” for convenient glucose detection (Figure 3D). The model achieved 99.64% accuracy (i.e., % recovery) in spiked serum samples (Table 1). Additionally, manual analysis of the sensor data resulted in a limit of detection (LOD) of 100 nm for glucose detection, while ML‐based analysis achieved an LOD of 10 nm. This work indicated the potential for combining an advanced electrode material with ML‐based analysis for achieving non‐enzymatic glucose sensing in complex biological samples.

Though at present, ML‐aided biocatalytic sensors have seen limited exploration, ML has nonetheless shown its usefulness in helping overcome variability in operating conditions and maintaining sensing performance despite decreasing enzyme activity. Though ML can predict the decrease in sensitivity of a glucose sensor, it remains to be seen if this can translate to more robust predictions in the presence of biofouling. This could prove helpful in alleviating user burden in CGMs, where ML could monitor the CGM sensitivity and account for it in glucose predictions, removing or reducing the need for user calibration. While more testing of these devices in complex biological fluids is necessary, a ML‐aided non‐enzymatic biocatalytic electrochemical sensor could achieve low LOD glucose detection in serum samples,^[^ ^78^ ^]^ showing the potential of these devices for use in real biological samples. The use of MoS_2_ to enable non‐enzymatic glucose sensing, along with the use of advanced/nanomaterials for increasing sensor response and enabling differentiation between different pesticides indicates the increasing relevance of biocatalytic advanced electrochemical biosensors. While biocatalytic sensors rely on enzyme‐ (or material‐) catalyzed reactions for biorecognition, direct recognition of binding events between an analyte and affinity elements (e.g., antibody or aptamer) is an alternative approach. Sensors employing this approach are known as affinity‐based and provide a viable alternative to enzyme‐based biorecognition.

### 3.2 Affinity‐Based Biosensors

Affinity‐based electrochemical biosensors make use of specific recognition elements to detect the analyte of interest. Recognition elements are typically immobilized on an electrochemical transducer. Binding of the analyte to the recognition elements can be transduced to a measurable signal that can be correlated to the analyte concentration.^[^ ^80^, ^81^, ^82^ ^]^ While antibodies have been commonly used as recognition elements, alternatives such as aptamers and molecularly imprinted polymers have been the subject of increasing interest.^[^ ^81^, ^82^, ^83^, ^84^, ^85^ ^]^ The relative merits and drawbacks of each type of affinity element have been discussed elsewhere.^[^ ^86^, ^87^, ^88^, ^89^, ^90^ ^]^ Pairing affinity‐based biorecognition with electrochemical transducers can allow for sensors with simple operation and high sensitivity.^[^ ^81^, ^91^, ^92^ ^]^ Advanced electrochemical affinity‐based sensors have been integrated with ML in various cases. ML has been used as a replacement for, or complement to, analytical circuit fitting for EIS, which is commonly used with affinity‐based sensors. Additionally, ML has been used to provide actionable predictions directly from electrochemical data, and to aid in sensor design.

**Figure 4:**
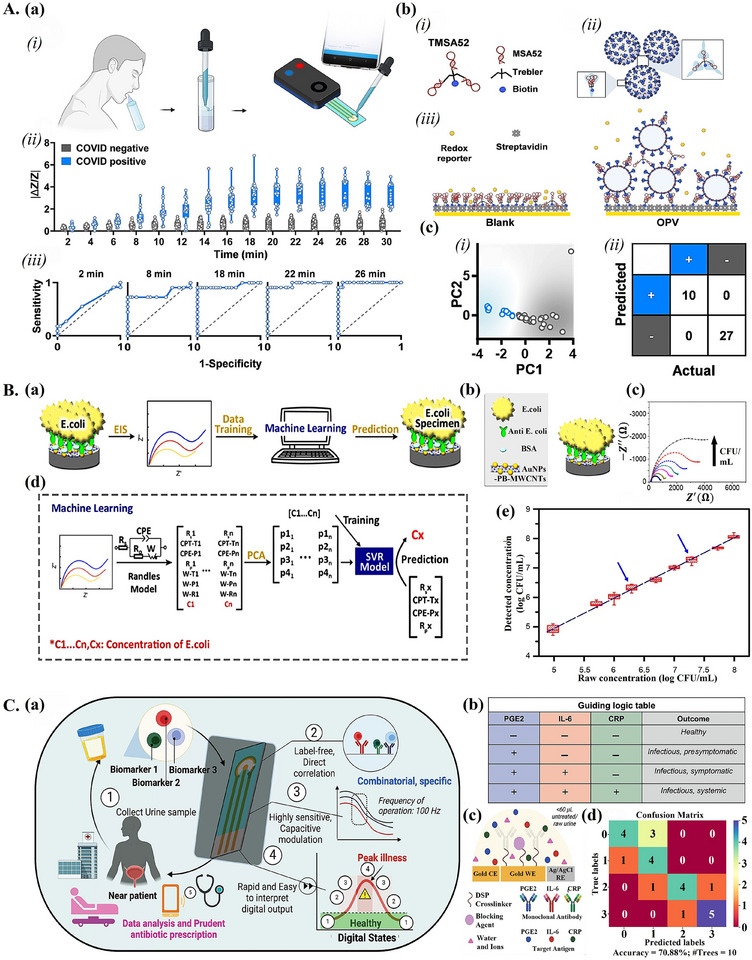
ML‐aided affinity‐based advanced electrochemical biosensors. A) ML‐aided electrochemical aptasensor for SARS‐CoV‐2 detection. a) Overview of device operation and performance. i) Sample collection by end‐user and transfer to device. ii) Time series impedance signal for COVID positive and negative samples, with the *y*‐axis showing normalized impedance (|Δ*Z*/*Z*|). iii) Receiver operating curves for samples from ii), at specific time points. b) i) Structure of trimeric TMSA52 aptamer for SARS‐CoV‐2 spike protein detection. ii) Binding of TMSA52 aptamer to SARS‐CoV‐2 spike protein. iii) Surface interactions on streptavidin‐modified working electrode surfaces for blank solutions (left) and solutions containing viral target (right). c) ML‐aided binary classification. i) Separation of SARS‐CoV‐2 positive (blue) and negative (grey) samples with two principal components. ii) Confusion matrix for SARS‐CoV‐2 detection on test set samples. a–c) Reproduced with permission.^[^ ^23^ ^]^ Copyright 2024, John Wiley and Sons. B) Advanced EIS affinity biosensor coupled with ML for E. coli detection. a) Workflow overview, including collection of EIS data used for ML model training. b) The electrode surface is modified with AuNPs‐PB‐MWCNTs, as well as bovine serum albumin (BSA) and anti‐*E. coli* antibody. c) EIS spectra over increasing bacterial concentration. d) Detailed ML workflow, including Randles model fitting, PCA, and SVR model training. e) Prediction on test set, including completely unseen (blue arrows) concentrations. a–e) Reproduced with permission.^[^ ^102^ ^]^ Copyright 2020, IOP Publishing. C) ML‐aided electrochemical affinity sensor for disease state classification of UTI. a) Overview of device operation. b) Guiding logic table for discrete UTI disease state prediction as a function of levels of the three biomarkers (PGE2, IL‐6, CRP). c) Overview of sensor electrodes and electrode surface functionalization. d) Confusion matrix for detection of the four digital disease states with RF. a–d) Reproduced with permission.^[^ ^34^ ^]^ Copyright 2022, John Wiley and Sons.

EIS is well‐suited for detecting bio‐recognition events occurring at the electrode surface,^[^ ^93^, ^94^, ^95^, ^96^, ^97^, ^98^ ^]^ and is therefore commonly used in affinity‐based electrochemical biosensors. However, analysis of EIS data requires complicated circuit fitting. Analysis of raw EIS data, or EIS time series, with ML can eliminate the need for circuit fitting and help maintain sensor detection performance in the presence of contamination and variability in samples or sensing conditions.^[^ ^3^ ^]^ For example, Sen et al.^[^ ^23^ ^]^ proposed a wash‐free, single‐pot ML‐aided aptasensor for SARS‐CoV‐2 detection based on single‐frequency EIS measurements. The assay workflow, shown in **Figure** **4**A,a(i), involved the transfer of saliva samples to a tube pre‐filled with assay reagents, followed by the transfer of the mixed solution to an electrochemical chip where normalized impedance (|Δ*Z*/*Z*|) could be measured via a smartphone‐interfaced potentiostat. A trimeric aptamer was assembled using monomeric MSA52 aptamers^[^ ^99^ ^]^ (Figure 4A,b(i)), specific to the spike protein of SARS‐CoV‐2 (Figure 4A,b(ii)), and was modified with biotin for linking to streptavidin‐modified electrodes (Figure 4A,b(iii)). Single‐frequency EIS measurements allowed for the collection of EIS time series for target binding events. Increased differences in impedance between COVID positive and negative samples could be seen over time (Figure 4A,a(ii)). Figure 4A,a(iii) shows receiver operating curves obtained for disease classification based on specific time points, showing an increase in sensitivity over time, coinciding with the increased difference in |Δ*Z*/*Z*| between positive and negative samples. Due to the time‐variance of sensitivity, the authors decided to analyze the whole time series with ML. Unsupervised learning was first used for curve fitting, extracting nine features from the impedance time series. These features were then reduced to 2D space using PCA (Figure 4A,c(i)) and fed to an SVM for classification. The model achieved 100% accuracy (Figure 4A,c(ii) and **Table** **2**). While stringent washing steps are typically considered necessary to eliminate sample‐to‐sample variability, the use of ML for analysis of time series allowed each sample to be considered relative to its own baseline, reducing the effect of variability and enabling simplification of the assay.

**Table 2:** Machine‐learning‐aided affinity‐based electrochemical biosensors.

Target	Materials	Fluid^a)^	EC method	ML type	Metrics	Year/Refs.
SARS‐CoV‐2	Screen‐printed gold WE	Saliva	Single‐frequency EIS	SVM	Acc. = 100%	2024^[^ ^23^ ^]^
*Ganoderma applanatum* agglutinin structure (sensor design)	GCE WE + PB films	N/A	N/A	AlphaFold	Confidence (pLDDT)^[^ ^108^, ^111^ ^]^ > 90%	2023^[^ ^107^ ^]^
Acetone	Pt/Ir WE	Water	EIS	SVM	Acc. = 95%	2018^[^ ^100^ ^]^
BNP	CNT‐TF electrodes	PBS, blood	EIS	DNN	Acc. (PBS) = 80%; Acc. (Blood) = 80%	2022^[^ ^101^ ^]^
*E. Coli*	MWCNTs/PB/AuNPs – modified WE	PBS	EIS	SVR	Average Prediction Error = 1.52 ± 0.136%	2020^[^ ^102^ ^]^
Amikacin Sulfate	WE modified with AuNPs‐modified nitrocellulose membrane	PBS	EIS	XGBoost	RMSE (2 h) = 0.0012 µL mL^−1^; RMSE (3 h) = 0.00102 µL mL^−1^; RMSE (4 h) = 0.00063 µL mL^−1^	2022^[^ ^103^ ^]^
Endometriosis	Screen‐printed gold WE modified with SAM	PBS, serum	EIS	Ensemble bagged trees	Acc. (PBS) = 99.8%; Acc. (Serum) = 99.5%	2021^[^ ^104^ ^]^
UTI endotype	Gold WE screen‐printed on ceramic substrate	Artificial urine	EIS	RF	Acc. = 70.88%	2023^[^ ^105^ ^]^
SARS‐CoV‐2 (incl. variants)	LSG – AuNP – modified WE	Nasopharyngeal swab	DPV	DNN	Acc. (Beta) = 98.7%; Acc. (Alpha) = 99.5%; Acc. (Delta) = 100%; Acc. (Control) = 99.37%	2022^[^ ^106^ ^]^

^a)^ Biological fluids are of human origin unless otherwise specified.

Variability can also be caused by weak interactions between the biorecognition element and the analyte, leading to differences between sensor replicates. Rong et al.^[^ ^100^ ^]^ proposed a ML‐aided impedimetric biosensor for acetone detection in the presence of sensor‐to‐sensor variability due to weak interactions between acetone and the chemosensory protein used for recognition. EIS data was directly processed with ML, removing the need for circuit fitting. PCA was used to reduce the EIS data to 2D space. An SVM was then trained with this data for binary acetone detection at clinically relevant levels (5 mm), achieving 95% accuracy (Table 2). In another study, deep‐learning‐based analysis of raw impedance spectra was compared to analytical circuit fitting.^[^ ^101^ ^]^ The proposed sensor aimed to diagnose heart failure through the detection of B‐type natriuretic peptide (BNP). A carbon nanotube‐thin film (CNT‐TF) electrode was functionalized with monoclonal anti‐BNP antibodies. Though CNT‐based biosensors show fast response and high sensitivity, their robustness is decreased by their inherent variability, ML analysis was introduced to help overcome this. A calibration curve for BNP concentration was first built on a single parameter (*Rcnt*: CNT‐TF resistance) extracted from equivalent circuit fitting of EIS data. This calibration curve was used to predict discrete BNP levels (four classes) on unseen data and achieved 39.9% accuracy. A deep neural network was then trained on 586 sets of raw EIS data representing 52 different BNP concentrations. This model achieved 80% accuracy on test samples, while further testing on patient whole blood samples also achieved a similar test accuracy of 80% (Table 2), surpassing the analytical approach. Thus, in affinity‐based electrochemical biosensors, direct analysis of impedance spectra with ML shows promise for overcoming weak affinity binding and can compensate for inherent variability in CNT‐TF sensors and outperform analytical circuit fitting.

In some cases, however, ML‐based analysis of parameters extracted from fitted EIS data may still be a worthwhile approach. Instead of building a calibration curve based on a single parameter, multiple extracted parameters can simultaneously be fed to a ML model. For instance, a machine‐learning‐assisted advanced affinity‐based EIS sensor for *E. coli* detection was designed.^[^ ^102^ ^]^ (Figure 4B,a). The sensor electrodes were coated with a nanocomposite material composed of multiwall carbon nanotubes (MWCNTs), Prussian blue (PB), and AuNPs, and were functionalized with *E. coli* antibody (Figure 4B,b). Corresponding increases in impedance were observed as *E. coli* concentrations increased (Figure 4B,c). Predictions of *E. coli* concentrations based on a single parameter (charge transfer resistance, *R_p_*) obtained by fitting a Randles model were found to be inaccurate. Instead, seven parameters extracted from circuit fitting were combined and reduced to four principal components using PCA. This dataset was then used to train a SVR model for *E. coli* concentration prediction (Figure 4B,d). The SVR model achieved an average test prediction error of 1.52%, with predicted concentrations corresponding well to actual values, including for two unknown concentrations that were introduced (Figure 4B,e, Table 2). In continuation of this work, Chen et al.^[^ ^103^ ^]^ used a similar approach for indirect detection of antibiotic concentration through bacterial proliferation. Electrodes modified with AuNP‐modified nitrocellulose membrane were functionalized with *E. coli* antibody for detection based on EIS. Seven parameters were extracted from EIS data based on Randles model fitting. Four out of seven were selected for training XGBoost and light gradient‐boosting machine (LightGBM) models. The XGBoost model performed best, achieving RMSEs of 0.0012, 0.00102, and 0.00063 µL mL^−1^ for amikacin sulfate antibiotic concentration detection at 2, 3, and 4 h after dosage, respectively (Table 2). While still requiring circuit fitting, analysis of multiple extracted EIS parameters via ML can outperform models based on single parameters and may be worthwhile in cases where expertise is available to extract relevant parameters from raw data.

While numerous affinity‐based electrochemical sensors may be capable of accurately and reliably quantifying a specific biomarker without the use of ML, interpretation of the information provided for a non‐expert may not be trivial, especially in the case of multiplexed sensors. Some ML‐aided affinity electrochemical biosensors have been designed to use ML to provide actionable diagnoses directly from electrochemical data. Pal et al.^[^ ^104^ ^]^ designed an affinity‐based advanced electrochemical sensor for endometriosis detection through the serum biomarker alpha‐1‐B glycoprotein (A1BG). Monoclonal anti‐A1BG antibody was used as an affinity element and was covalently immobilized on a self‐assembled‐monolayer (SAM) modified screen‐printed electrode. Analytical fitting of EIS data allowed for the detection of A1BG based on a calibration curve. Testing in serum samples from healthy and endometriotic patients showed good agreement between the proposed sensor and gold standard ELISA for A1BG quantification. ML was then implemented for direct differentiation between healthy and endometriotic samples (binary classification) from impedance data. An ensemble bagged trees model, trained on impedance data collected from samples with healthy and endometriotic concentrations of A1BG, was found to perform best. In spiked PBS samples, the model achieved an overall accuracy of 99.8%, while in patient serum samples, the accuracy was of 99.5% (Table 2). While the simplicity of a binary output is appealing, classification into strata of disease severity has also been explored. A machine‐learning‐based workflow entitled “DigEST” was proposed to digitize electrochemical biosensor output.^[^ ^105^ ^]^ The concept was demonstrated for the detection of urinary tract infection (UTI), where the output was in the form of predicted disease endotype, to provide actionable and interpretable information for the user. The device workflow (Figure 4C,a) involved EIS analysis of collected urine by ML, with the output of a digital value corresponding to the disease state (1 to 4). Three UTI‐associated biomarkers; prostaglandin E2 (PGE2), interleukin‐6 (IL‐6), and C‐reactive protein (CRP), were chosen. Digital disease states were defined according to levels of these biomarkers (Figure 4C,b). Monoclonal antibodies for the three markers were used to functionalize the sensor working electrode (Figure 4C,c). A RF model was trained on impedance data and achieved 70.88% accuracy (Figure 4C,d and Table 2) for the detection of the different disease states. Though the model performance showed room for improvement, this work demonstrated proof‐of‐concept for a workflow applicable to other diseases requiring stratification of severity. Aside from the classification of disease endotypes, the identification of disease strains is also useful. Beduk et al.^[^ ^106^ ^]^ designed an advanced electrochemical affinity sensor for the detection of SARS‐CoV‐2 variants. A laser‐scribed graphene (LSG) sensor, coupled with gold nanoparticles, was functionalized with ACE2 receptor for detection of SARS‐CoV‐2 spike protein (S1/S2 protein) and integrated with a smartphone‐operated portable potentiostat. A deep neural network was trained on DPV data to detect variants B.1.1.7 (alpha), B.1.351 (beta), and B.1.617.2 (delta), as well as control patients (healthy). The neural network achieved 98.7% accuracy for the beta variant, 99.5% accuracy for the alpha variant, 100% accuracy for the delta variant, and 99.37% for control patients (Table 2). Thus, while a traditional electrochemical sensor typically produces a numerical value for analyte concentration, leaving interpretation of this value up to the user, an ML‐aided electrochemical sensor can provide more interpretable outputs, whether it be a binary disease prediction, stratification into disease states, or disease strain classification.

Aside from the analysis of electrochemical data from affinity sensors, ML can also aid in affinity sensor design. Abrantes‐Coutinho et al.^[^ ^107^ ^]^ proposed an affinity‐based electrochemical glucose sensor designed with the help of ML. *Ganoderma applanatum* lectin (GAL), a fungal lectin showing affinity for glucose, was used as a biorecognition element. As GAL's structure was not known, strategies for immobilization on the electrode surface could not be identified. Therefore, DeepMind's AlphaFold, a neural‐network‐based model for the prediction of 3D protein structures from amino acid sequences,^[^ ^108^ ^]^ was used to elucidate the structure of a *Ganoderma applanatum* agglutinin, used as a GAL homolog. The structure was predicted with a high confidence score (>90%) (Table 2) and indicated a predominance of negatively charged domains with the presence of some positively charged domains. Based on this information, it was determined that a Prussian‐blue‐modified glassy carbon electrode (GCE) would be suitable for GAL immobilization. In testing of the designed sensor for glucose quantification in pharmaceutical formulations (intravenous replacement solution), recovery levels of 98.8–100.1% were achieved. The use of ML therefore enabled elucidation of the unknown protein structure, facilitating the choice of electrode immobilization strategy.

In summary, advanced affinity electrochemical biosensors have made use of various advanced/nanomaterials, including CNTs, AuNPs, and LSG, and have benefited from ML integration in various ways. ML has aided in the analysis of EIS data, in providing interpretable and actionable outputs directly from electrochemical data, and in electrode design. In the future, ML‐enabled analysis of raw EIS data may become increasingly common, as this simplifies sensor data analysis workflows and may identify subtleties in raw data that might be lost with parameters extracted from analytical fitting. The use of ML to provide interpretable and actionable outputs may become increasingly relevant, especially as multiplexed affinity sensors become more common.^[^ ^109^ ^]^ Though the use of a specific biorecognition element provides increased specificity for the analyte of interest, sensors requiring a biorecognition element are not free of disadvantages.^[^ ^6^, ^110^ ^]^ With the aim of overcoming these disadvantages, recent work has also investigated bioreceptor‐free electrochemical biosensors.

### 3.3 Bioreceptor‐Free Biosensors

Bioreceptor‐free biosensors have emerged to overcome the stability and degradation issues of bioreceptor‐based sensors, as well as to remove the need for complex and expensive biomolecule synthesis.^[^ ^110^ ^]^ As opposed to a typical electrochemical sensor, bioreceptor‐free electrochemical sensors make use of un‐modified electrodes with no immobilized biorecognition element to detect the analyte. Without a biorecognition element, it may be challenging to maintain strong specificity and a low LOD in complex mixtures, such as clinical samples. ML can be introduced to improve performance through modeling and its ability to detect subtle patterns in sensor responses.^[^ ^6^ ^]^ More specifically, ML has been paired with bioreceptor‐free advanced electrochemical biosensors to resolve components with similar electrochemical responses on unmodified electrodes, to analyze analyte fingerprints generated from electrochemical sensor arrays (i.e., electrochemical tongue), and to deal with electrode fouling.

**Figure 5:**
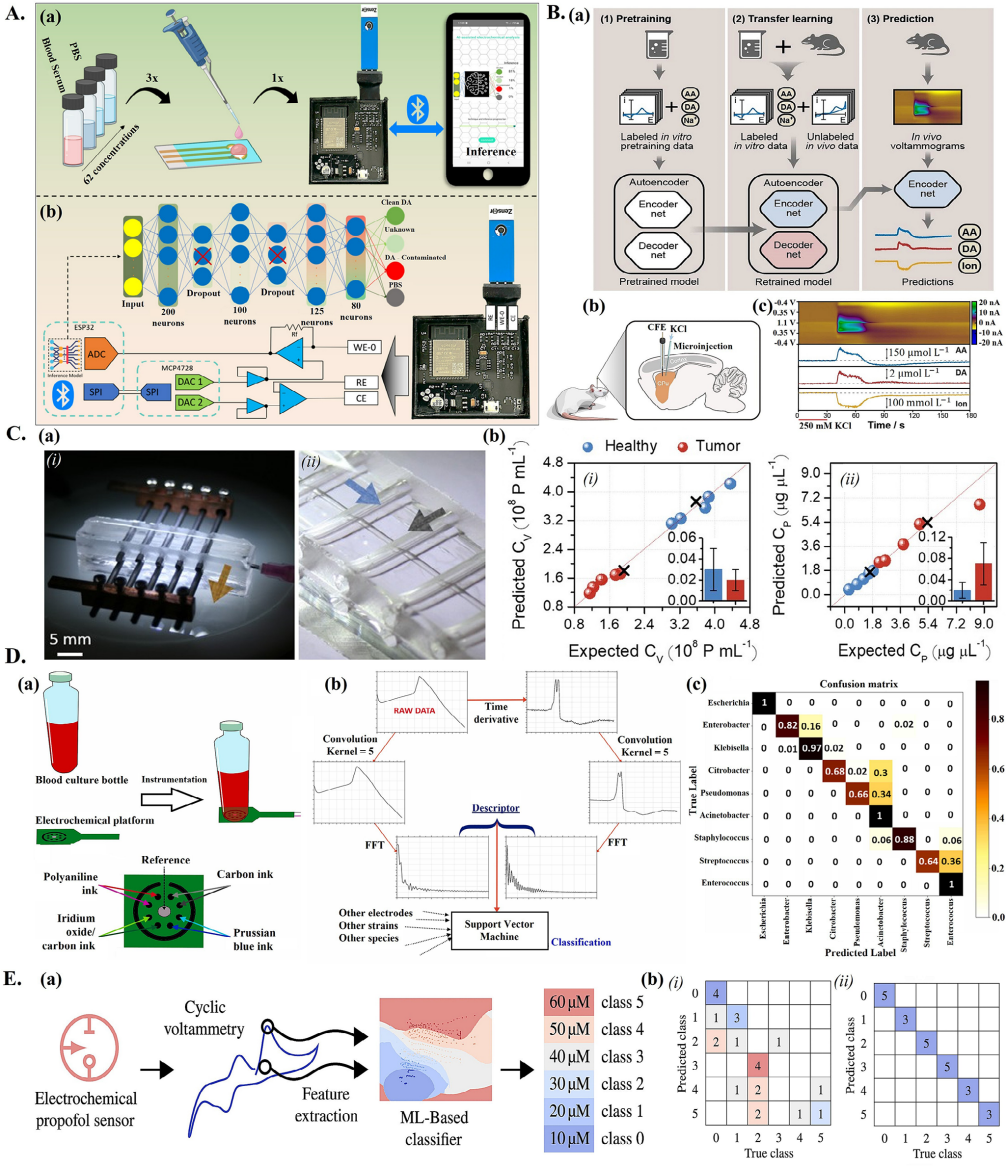
ML‐aided advanced bioreceptor‐free electrochemical biosensors. A) ML for bioreceptor‐free detection of dopamine. a) Overview of ML‐assisted, smartphone‐coupled, portable potentiostat for dopamine detection. b) Structure of neural network used for sample classification (clean versus contaminated) and block diagram of custom‐made potentiostat circuit. a,b) Reproduced with permission.^[^ ^21^ ^]^ Copyright 2023, John Wiley and Sons. B) ML‐aided advanced bioreceptor‐free sensing in living animal brain. a) Overview of ML workflow. b) Overview of in vivo experiment set‐up: implanted carbon‐fiber electrode, potassium chloride (KCl) administration for induction of spreading depression. c) Top: color plot for voltammograms of KCl‐induced spreading depression. Bottom: concentration predictions for AA, DA, and ionic charges, as calculated by ML model. a–c) Reproduced with permission.^[^ ^115^ ^]^ Copyright 2021, John Wiley and Sons. C) Extracellular vesicle characterization with ML‐paired impedimetric electronic tongue. a) Device overview. i) PDMS microfluidic chip with electrodes inserted transversally. ii) Microfluidic channel (black arrow) intersecting electrode channels (blue arrow). b) RF predictions for i) *C_v_* and ii) *C_P_*, with inset showing average confidence intervals for the respective variable. a,b) Reproduced with permission.^[^ ^110^ ^]^ Copyright 2023, Springer Nature. D) ML‐aided electrochemical pathogen sensor integrated with a blood culture bottle. a) Overview of ML‐aided smart blood culture bottle (top), with custom electrochemical platform (bottom), for pathogen identification and bloodstream infection diagnosis. b) Overview of data pre‐processing and analysis pipeline. c) Confusion matrix for identification of each pathogen genus. a–c) Reproduced with permission.^[^ ^122^ ^]^ Copyright 2023, Elsevier. E) ML to overcome electrode fouling in bioreceptor‐free propofol monitoring. a) Overview of proposed workflow, with ML‐based discrete propofol quantification. b) Results in human serum. i) Confusion matrix for standard linear model. ii) Confusion matrix for proposed SVM. a,b) Reproduced with permission.^[^ ^30^ ^]^ Copyright 2021, Elsevier.

In electrochemical sensors with unmodified electrodes, it may be difficult to resolve analytes that have similar or interfering electrochemical responses, ML can detect subtleties in electrochemical signals that can allow such analytes to be resolved. In one study, the authors proposed a custom‐made, bioreceptor‐free electrochemical sensor, making use of a screen‐printed carbon electrode, and paired with a portable potentiostat, controllable from a smartphone app. AI was integrated for POC dopamine detection aiding in overcoming interference of other species present in cerebrospinal fluid, such as uric acid (UA) and ascorbic acid (AA, **Figure** **5**A,a). To enable a “portable” ML model, the TinyML framework, which aims to “miniaturize” ML models, allowing ML inference on low‐power hardware,^[^ ^112^ ^]^ was implemented. The TinyML model was trained with square‐wave voltammetry (SWV) curves and integrated into the custom‐made potentiostat. The model classified samples into either clean DA, contaminated DA, PBS, or unknown (Figure 5A,b). SWV curves for DA, AA, and UA were found to overlap; ML was implemented to help resolve these overlapping signals. Samples labeled as clean DA could be quantified using a calibration curve, while the detection of contaminated DA indicated the need for further cleaning or the use of a different calibration curve. Quantization was used to reduce the model size to allow it to be run on the portable potentiostat. The model achieved a maximal accuracy of 96.01% after 8‐bit quantization (**Table** **3**). Further testing with human blood serum samples showed that the model could successfully identify contaminants. This study demonstrated the potential for the use of ML in low‐power portable systems useful in low‐resource applications allowing data processing in real time and with increasing reliability.^[^ ^21^ ^]^ Another study quantified dopamine, serotonin, AA, and UA in complex mixtures using an electrode modified with electrochemically reduced graphene oxide (ERGO), coupled with CV and ML for analysis. Electrode modification with ERGO was employed to improve the electrochemical signal. CV data was compressed by 73.3% with discrete wavelet transform prior to training a shallow FCNN. The model achieved an *R* ^2^ of 0.9744, 0.9794, 0.9784, and 0.9847 on the test set, for quantification of DA, serotonin, AA, UA, respectively (Table 3). Total RMSE on the training set was of 0.55 µM while the total RMSE on the test set was of 5.37 µm, indicating some overfitting, likely due to the use of only 45 samples for training and testing.^[^ ^113^ ^]^ Aside from the detection of DA, bioreceptor‐free electrochemical biosensors have also been applied for the detection of bacterial contamination. In one study, researchers proposed a bioreceptor‐free impedance‐based biosensor capable of detecting three different bacterium types, namely *Escherichia coli* strains JM109 and DH5‐α, and *Salmonella typhimurium*. The sensor consisted of inter‐digital silver electrodes with silver nanowires uniformly decorated on the electrodes, this was intended to increase the electrode sensitivity for detection of bacteria. This approach utilized impedance measurements from linear sweep voltammetry (LSV), processed and classified using linear discriminant analysis (LDA), linear maximum likelihood estimation (MLE), and FCNN, all achieving 100% classification accuracy (Table 3). The features analyzed included power, current–voltage curve, and the first and second derivatives of the current–voltage characteristics.^[^ ^114^ ^]^ Combined analysis of these features by ML models allowed the sensor to distinguish between the different types of bacteria on an unmodified electrode. Overall, ML shows potential for resolving interfering substances on unmodified electrodes.

**Table 3:** Machine‐learning‐aided bioreceptor‐free electrochemical biosensors.

Target	Materials	Fluid^a)^	EC Method	ML Type	Metrics	Year/Refs.
Dopamine	Screen‐printed carbon WE	PBS, serum	SWV	FCNN (TinyML)	Acc. (PBS) = 96.01%	2023^[^ ^21^ ^]^
Dopamine, Serotonin, AA, UA	Graphite epoxy composite WE modified with ERGO	PBS	CV	FCNN	Dopamine *R* ^2^ = 0.9744; Serotonin *R* ^2^ = 0.9794; AA *R* ^2^ = 0.9784; UA *R* ^2^ = 0.9847	2019^[^ ^113^ ^]^
*Salmonella typhimurium, Escherichia coli (*strains JM109, DH5‐***α***)	Inter‐digital silver WE modified with silver nanowires	Water	LSV, Impedimetric	Linear MLE, LDA, FCNN	Acc. (MLE, LDA, FCNN) = 100%	2018^[^ ^114^ ^]^
Dopamine, Ascorbate, NaCl	VGCFE WE	aCSF and live rat brain tissue	FSCV	Convolutional autoencoder	(Figure 5B,c)	2021^[^ ^115^ ^]^
Synaptic Dopamine Release	Carbon fiber and stainless steel WEs	Live mouse/Rat brain tissue	FSCV	SVM, CNN	Acc. (SVM) = 79.15 ± 0.67%; Acc. (CNN) = 97.35± 5.84%	2019^[^ ^116^ ^]^
Oral cavity cancer	304 stainless steel microwire electrodes modified with SiO_2_, NiO_2_, Al_2_O_3_, and Fe_2_O_3_ oxide films	Saliva	Impedimetric	SVM, RF	Binary cancer detection acc. (SVM) = 86.7%; Cancer type detection acc. (RF) = 66.7%	2022^[^ ^118^ ^]^
Extracellular Vesicle concentration (***C***_***V***_) and carried proteins (***C***_***p***_)	Graphite core electrodes	PBS	Potentiometry Impedimetric	RF	*R* ^2^(*C_V_*); > 0.99; MAE (*C_V_*); = 0.1 × 10^8^ *P*/*mL*; *R* ^2^(*C_p_*) > 0.92; MAE (*C_p_*) = $0.7\frac{\mu g}{\mu L}$	2020^[^ ^121^ ^]^
Propofol	Pencil lead WE	PBS, serum	SCV	SVM	Acc. (PBS) = 98.9%; Acc. (Serum) = 100%	2021^[^ ^30^ ^]^
Blood pathogen Gram and genus identification	Different material WEs (polyaniline ink, carbon ink, iridium oxide/carbon ink, Prussian blue ink)	Blood	Potentiometry	SVM	Acc. (Gram) > 95%; Acc. (genus) < 80%	2023^[^ ^122^ ^]^

^a)^ Biological fluids are of human origin unless otherwise specified.

The studies above have mostly limited themselves to “clean” samples (i.e., water, PBS) that may not be representative of the noise and interference present in real biological samples. Some researchers have, however, applied ML‐aided bioreceptor‐free advanced electrochemical sensors for in vivo testing. For instance, Xue et al.^[^ ^115^ ^]^ proposed a deep‐learning‐aided voltametric sensing platform for in vivo neurotransmitter and ion sensing in living rat brain. The sensor made use of a vapor‐grown carbon fiber microelectrode (VGCFE), which contributed to generating stable and reproducible voltammograms. To predict concentrations of DA, ascorbate (AA), and NaCl from fast‐scan cyclic voltammetry (FSCV) data, an autoencoder with convolutional encoder and decoder was used. This model was first pre‐trained using labeled in vitro data, collected from artificial cerebrospinal fluid (aCSF), it was then fine‐tuned in a semi‐supervised manner with a mix of labeled in vitro data and unlabelled in vivo data (collected from living rat brain striatum), and finally tested for measurement of DA, AA, and ion concentrations under spreading depression in living rat brain striatum (Figures 5B,a–c). Spreading depression is a phenomenon of particular interest in the study of brain function and disease, and the concentration time series predicted by the encoder was consistent with previously reported reaction‐diffusion mechanisms of spreading depression propagation. This work represents an interesting example of pre‐training, which, in settings where in vivo data availability is limited, can be used to improve model performance by pre‐training with in vitro data.^[^ ^115^ ^]^ In another work,^[^ ^116^ ^]^ a bioreceptor‐free electrochemical sensor using FSCV was paired with a CNN for binary classification of synaptic DA release in live mice and rat brain. The CNN was used for analysis of FSCV images; it was compared with SVM, which was trained with features manually extracted from FSCV data. The CNN achieved an accuracy of 97.35% for binary classification of DA release while SVM achieved an accuracy of 79.15% (Table 3), indicating that the features extracted automatically by the CNN may have been better predictors of DA release than those extracted manually. Thus, ML‐aided bioreceptor‐free electrochemical sensors have also shown good performance when tested in live tissue.

As an alternative to a single electrode, electrode arrays have been used to generate unique analyte “fingerprints”, in an arrangement known as “electronic tongue”, or e‐tongue. Coupled with ML, these fingerprints can enable specific analyte detection without requiring a biorecognition element.^[^ ^117^ ^]^ One paper reports on the use of an e‐tongue based on impedance spectroscopy to detect oral cavity cancer,^[^ ^118^ ^]^ a type that is difficult to diagnose in its early stages^[^ ^119^, ^120^ ^]^ with saliva samples from diagnosed patients. The device consisted of 304 stainless steel microwires, modified with 800 nm of SiO_2_, NiO_2_, Al_2_O_3_, and Fe_2_O_3_ oxide films through electron beam deposition, and embedded in PDMS. Saliva from 27 cancerous patients and 14 controls was used. SVM with RBF kernel achieved an accuracy of 86.7% for binary detection of cancer, while RF achieved an accuracy of 66.7% for detection of the cancer type (Table 3). This study also demonstrated that including clinical information (e.g., alcohol consumption) in conjunction with the e‐tongue data further increased the accuracy.^[^ ^118^ ^]^ In another publication,^[^ ^121^ ^]^ researchers demonstrate that low‐cost, fast, accurate, and scalable diagnostics can be achieved by converging an impedimetric electronic tongue with ML for providing the simultaneous determination of two extracellular vesicle (EV) biomarkers: EVs concentration (*C_V_*) and carried proteins (*C_P_*). The proposed device was a simple PDMS microfluidic chip with multiple integrated graphite electrodes (Figure 5C,a). Both *C_V_* and *C_P_* could be attained from a single impedance spectrum with the aid of ML. Supervised sure independence screening and sparsifying operator (SISSO) and RF models were tested, with RF showing better predictive performance for *C_V_* (*R* ^2^ > 0.99) and *C_P_* (*R* ^2^ > 0.92) (Figure 5C,b and Table 3). Electrode arrays have also been used for detection of bloodstream infections; Babin et al.^[^ ^122^ ^]^ explored the use of potentiometric measurements in blood cultures with a multi‐material electrode array integrated into a blood culture bottle (Figure 5D,a). The electrode array was formed through ink deposition using polyaniline, carbon, iridium oxide/carbon, and Prussian blue inks. This portable system detected pathogen genus and Gram type by generating specific potential curve fingerprints unique to 14 species and 9 genera. After applying smoothing and fast Fourier transform, these data were used to train an SVM model (Figure 5D,b), achieving up to 99% accuracy for Gram classification and up to 85% accuracy for genus identification^[^ ^122^ ^]^ (Figure 5D,c and Table 3). This approach shows promise for rapid and decentralized diagnosis of positive blood cultures without complex handling of the contaminated sample. In applying ML to data obtained from electronic tongues, or electrochemical sensor arrays, caution is necessary, as the sensing data obtained can have considerable width (large number of features), which may result in overfitting,^[^ ^123^, ^124^ ^]^ especially with smaller datasets where the number of features will be larger than the number of samples.

Though less specific to bioreceptor‐free sensors than lack of specificity, electrode fouling remains an important concern, especially in continuous therapeutic drug monitoring (TDM), which is essential for optimizing drug dosage and preventing inadequate sedation and its associated complications.^[^ ^125^, ^126^ ^]^ Most sensors for monitoring the intravenous anesthetic propofol at sufficiently low LODs are single‐use^[^ ^127^ ^]^ due to electrode fouling caused by the formation of a polymeric film,^[^ ^128^ ^]^ making them unsuitable for automated TDM in a closed‐loop system To address this, ML was introduced for discrete quantification of propofol in the presence of fouling (Figure 5E,a). Staircase cyclic voltammetry (SCV), paired with an electrochemical cell making use of pencil lead electrodes, was used to collect data in PBS and undiluted human serum. Fouling was found to lower the current peak in SCV data collected over repeated measurements. An RBF‐SVM was trained on a dataset of 480 samples and tested on 120 samples, achieving a classification accuracy of 98.9% in PBS and 100% in undiluted human serum, outperforming a linear model (Figure 5E,b, Table 3). This method allows detection at therapeutic concentrations between 1 and 60 µm, with a focus on the 10 µm level, for up to 10 min with sampling every 30 s, enabling more precise and safer sedation through live and continuous monitoring.^[^ ^30^ ^]^

Bioreceptor‐free advanced electrochemical sensors have made use of various advanced/nanomaterials, including ERGO, silver nanowires, and VGCFE, which were mainly used to increase sensitivity and improve the electrochemical signal. Bioreceptor‐free advanced electrochemical sensors have also benefited from ML inclusion. ML was used to help eliminate the need for a specific biorecognition element by resolving interfering electrochemical signals from unmodified electrodes, and by analyzing fingerprint‐like data obtained from an electrochemical sensor array. ML could also help overcome electrode fouling. Testing in serum samples and in live brain tissue has indicated that ML‐aided bioreceptor‐free advanced electrochemical sensors can perform well with complex biological samples, though further testing will be necessary to truly confirm the robustness of these sensors. With electrode arrays, caution will be necessary to avoid overfitting due to wide data. While the approaches discussed so far have made use of purely electrochemical transduction, producing an electrical signal, electrochemical detection can also be paired with chemiluminescence, in the form of electrochemiluminescence, producing a visual signal, and such sensors can also benefit from ML‐based analysis.

### 3.4 Electrochemiluminescence‐Based Biosensors

Electrochemiluminescence is an electrochemical phenomenon wherein species are generated at the electrode surface and undergo electron‐transfer reactions, forming excited states that emit light.^[^ ^129^, ^130^, ^131^ ^]^ The light‐emitting species are known as luminophores and become activated upon application of the proper voltage.^[^ ^132^ ^]^ Readers interested in ECL fundamentals are referred to previous reviews.^[^ ^131^, ^133^ ^]^ ECL‐based biosensors rely on the correlation between the emitted light and the target analyte concentration to detect and quantify the target.^[^ ^132^, ^133^ ^]^ Such sensors have been increasingly used for the detection of various disease‐related biomarkers due to their versatility, high controllability, and wide detection range with low background signal and high sensitivity.^[^ ^129^, ^130^, ^132^, ^133^, ^134^, ^135^ ^]^ However, challenges to the wider use of ECL biosensors remain and share similarities with those faced by other types of biosensors. Namely, there is room for improvements in sensitivity and specificity, and for further miniaturization and increase in portability, especially for POC applications.^[^ ^134^, ^136^, ^137^ ^]^ Unfortunately, sensor miniaturization can lead to decreasing sensor reliability,^[^ ^138^, ^139^ ^]^ there is thus room in ECL‐based sensors for integration of ML to improve sensor sensitivity and specificity, and to enable miniaturization while maintaining sensor reliability.

**Figure 6:**
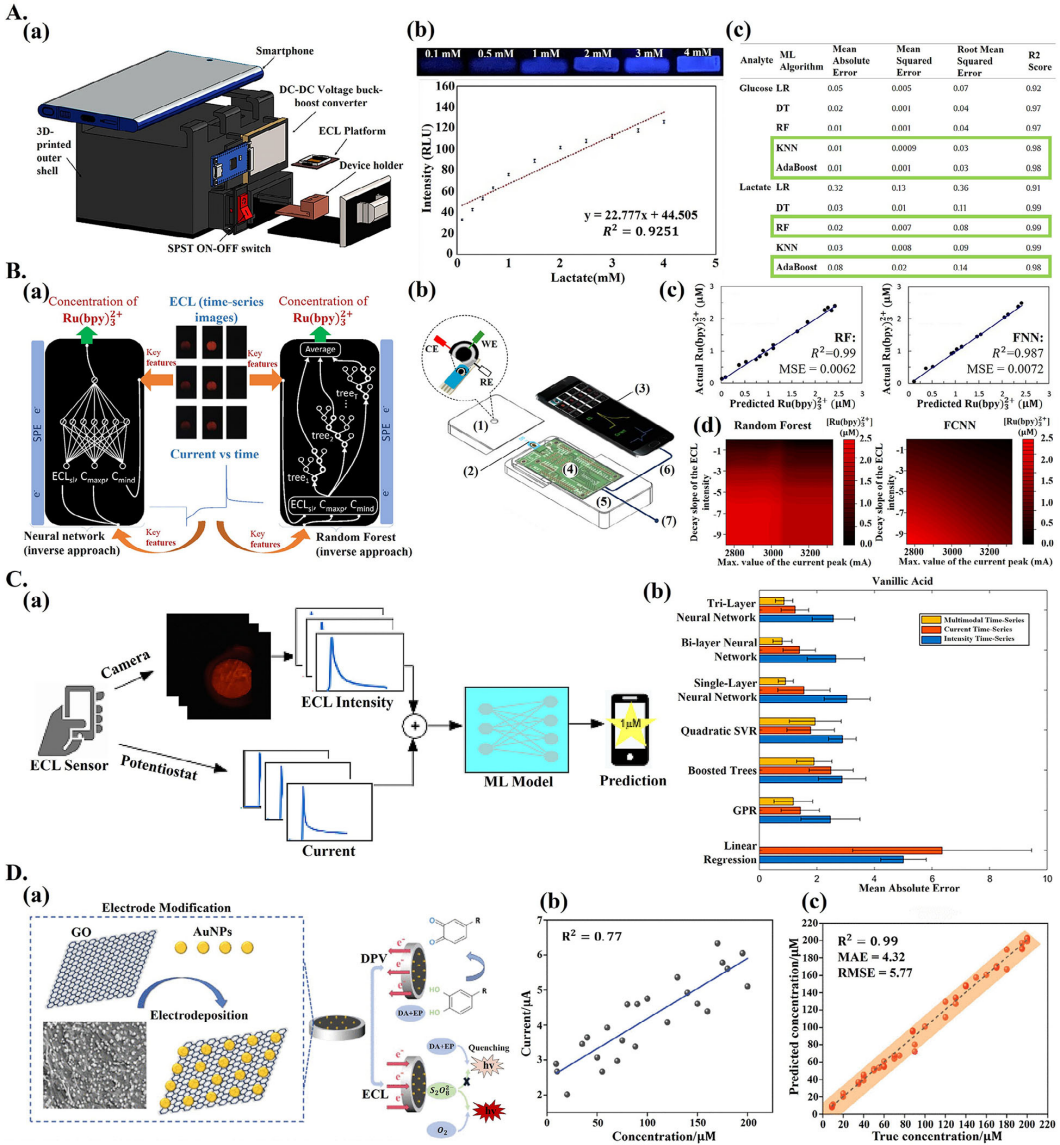
ML integration with advanced ECL‐based biosensors. A) ML‐aided ECL‐based detection of glucose and lactate. a) Illustration of 3D‐printed ECL platform with key components labeled. b) Top: Increasing ECL intensity with increasing lactate concentration. Bottom: ECL sensor calibration curve for lactate. c) Performance of liner regression, DT, RF, k‐nearest neighbors, and AdaBoost ML models for glucose and lactate detection, with best performers in green boxes. a–c) Reproduced with permission.^[^ ^140^ ^]^ Copyright 2023, Elsevier. B) ML‐aided ECL‐amperometric sensor for luminophore detection. a) Training of FCNN and RF models with key features extracted from ECL time‐series images and chronoamperometric data. b) Overview of the smartphone‐based device, including magnifying lens (1), screen‐printed electrodes (2), smartphone (3), potentiostat circuit (4), light‐tight container (5), USB cable (6), and power cable (7). c) Validation set performance of RF (left) and FNN (right) for $Ru{\left(bpy\right)}_{3}^{2+}$ concentration detection. d) Contour maps showing importance of ECL and amperometry data features on predicted $Ru{\left(bpy\right)}_{3}^{2+}$ concentration for RF (left) and FNN (right) models. a–d) Reproduced with permission.^[^ ^145^ ^]^ Copyright 2020, MDPI. C) ML‐aided ECL‐amperometric sensor for phenolic compound detection. a) Overview of sensor workflow. b) Performance of various ML models for VA quantification based on multimodal and single‐modal time series data. a,b) Reproduced with permission.^[^ ^139^ ^]^ Copyright 2021, MDPI. D) ML‐aided advanced ECL‐DPV biosensor for dopamine detection. a) Electrode modification with graphene oxide and gold nanoparticles, followed by electrode use for DPV and ECL data collection. SEM image of GO/AuNPs/GCE shown in bottom left corner. b) Prediction curve based on DPV data in the presence of EP interference. c) Test set performance for PSO‐ANN Dopamine quantification. a–c) Reproduced with permission.^[^ ^146^ ^]^ Copyright 2024, IEEE.

For instance, Kumar et al.^[^ ^140^ ^]^ proposed ML‐aided, ECL‐based detection of glucose and lactate. A portable, 3D‐printed, ECL sensor making use of a smartphone for imaging (**Figure** **6**A,a), paired with a custom smartphone app, was designed. The sensor made use of laser‐induced graphene (LIG) electrodes. The sensor calibration curve showed good linearity (Figure 6A,b), however, Adaboost and RF ML models allowed for more accurate glucose and lactate concentration prediction with *R* ^2^ of 0.98 and 0.99, respectively (Figure 6A,c and **Table** **4**). The successful pairing of ML with ECL for glucose and lactate detection indicated the potential of the portable device for the detection of other human metabolites. Whereas this work used unimodal ML, analyzing only ECL data, other researchers have investigated combinations of ECL data with other electrochemical data modalities. Such multi‐modal configurations can improve the accuracy and reliability of biosensors,^[^ ^141^, ^142^, ^143^ ^]^ and can be facilitated by ML,^[^ ^144^ ^]^ As an example, Ccopa Rivera et al.^[^ ^145^ ^]^ proposed a smartphone‐based multi‐modal sensor for $Ru{\left(bpy\right)}_{3}^{2+}$ luminophore detection. RF and FCNN models were trained to predict $Ru{\left(bpy\right)}_{3}^{2+}$ concentration ($\left[Ru{\left(bpy\right)}_{3}^{2+}]\right)$ from ECL and chronoamperometry data (Figure 6B,a). The sensor was paired with a smartphone for the capture of ECL images and made use of a portable potentiostat circuit for chronoamperometry (Figure 6B,b). Both trained models performed well on the validation set (Figure 6B,c) and achieved the test set *R* ^2^ values of 0.996 (RF) and 0.961 (FNN) for luminophore concentration prediction (Table 4). Contour plots (Figure 6B,d) were created to show the relationship between the input variables, *C_maxp_* (current max. peak) and *ECL_sl_* (ECL decay slope), and the predicted $\left[Ru{\left(bpy\right)}_{3}^{2+}\right]$. These plots indicated higher dependence of $\left[Ru{\left(bpy\right)}_{3}^{2+}\right]$ on the value of *ECL_sl_* than that of *C_maxp_*, helping to improve interpretability of the proposed models. In further work by the same group,^[^ ^139^ ^]^ an ML‐aided ECL‐chronoamperometry sensor for phenolic compound (vanillic acid‐VA and *p*‐coumaric acid‐pCA) detection was proposed (Figure 6C,a). Various ML models were trained with ECL–chronoamperometry time series or with features extracted from time series. Model performance was evaluated for uni‐ and multi‐modal data configurations. The authors found a high amount of variability in their data, due to sensor‐to‐sensor variations and changes in operating conditions and observed that the proposed ML models outperformed traditional calibration curves in the presence of such variability. For VA detection, the neural network model achieved an *R* ^2^ of 0.863 on time series data (Table 4). For pCA detection, Gaussian process regression (GPR) performed best with *R* ^2^ of 0.864 on extracted features (Table 4). No clear performance advantage was observed in favor of extracted features or time series, while multi‐modal configurations showed performance improvements over uni‐modal configurations, especially for neural network models (Figure 6C,b). In a similar vein, Rao et al.^[^ ^146^ ^]^ developed a multi‐modal advanced ECL‐DPV biosensor paired with ML for the detection of dopamine under epinephrine (EP) interference. Electrodes were modified with electrodeposited graphene oxide (GO) and gold nanoparticles to enhance the sensitivity and stability of the sensor (Figure 6D,a) and were used for the collection of DPV and ECL data for mixed DA/EP solutions. Calibration‐curve‐based DA detection was poor in the presence of EP interference (Figure 6D,b). ML was applied to help resolve DA; an FCNN was trained on ECL‐DPV data using the biologically inspired particle swarm optimization (PSO) algorithm. Using PSO‐FCNN, an *R* ^2^ value of 0.99 for dopamine concentration prediction was achieved on the test dataset, with an RMSE of 5.77 µm (Figure 6D,c and Table 4). The authors also found improvements in performance when using multi‐modal vs uni‐modal data and found performance to improve as more features were fed to the ML model. Testing of the predictive model on real samples (goat serum and artificial urine) showed relative standard deviation (RSD) values ranging from 1.57% to 9.25%. ML‐based fusion of additional data modalities with ECL data has resulted in performance improvements over approaches based on a single data modality; this approach has helped resolve interfering samples and has helped achieve improved performance over traditional calibration curves in the presence of variability in the data. Still, integration of ML with ECL‐based sensors has remained limited, and more testing, especially in more biologically relevant samples, will be needed. While the studies reviewed so far have extracted features from ECL data, direct analysis of ECL images enabled by ML could simplify the process, as exemplified in colorimetric sensing.^[^ ^147^, ^148^ ^]^ Increasing miniaturization and portability remains a continuing concern, not only with ECL‐based biosensors,^[^ ^136^, ^137^ ^]^ but with electrochemical biosensors in general,^[^ ^149^, ^150^ ^]^ especially in applications such as high‐throughput sensing and continuous monitoring.

**Table 4:** ML‐aided electrochemiluminescence‐based biosensors.

Target	Materials	Fluid^a)^	EC method	ML type	Metrics	Year/Refs.
Glucose, lactate	LIG WE	Water	ECL	Adaboost, RF	Glucose (Adaboost):; RMSE = 0.03 mm; *R* ^2^ = 0.98; Lactate (RF):; RMSE = 0.08 mm; *R* ^2^ = 0.99	2023^[^ ^140^ ^]^
$\mathrm{Ru}{\left(\mathrm{bpy}\right)}_{3}^{2+}$ luminophore	Screen‐printed carbon WE	PBS	ECL, Chronoamperometry	RF	RF:; MSE = 0.0012 µm^2^; *R* ^2^ = 0.996	2020^[^ ^145^ ^]^
Phenolic compounds	Screen‐printed carbon WE	PBS	ECL, Chronoamperometry	FCNN, GPR	VA (FCNN):; RMSE = 1.410 µm; *R* ^2^ = 0.863; pCA (GPR):; RMSE = 1.635 µm; *R* ^2^ = 0.864	2021^[^ ^139^ ^]^
Dopamine	GCE WE modified with reduced GO – AuNPs	PBS, Goat serum, artificial urine	ECL, DPV	PSO – FCNN	RMSE (PBS) = 5.77 µm; *R* ^2^ (PBS) = 0.99;	2024^[^ ^146^ ^]^

^a)^ Biological fluids are of human origin unless otherwise specified.

### 3.5 High‐Throughput Biosensors

High‐throughput biosensors are characterized by an ability to simultaneously (or nearly simultaneously) detect a relatively large number of analytes in one or multiple samples, or single or multiple analytes in a relatively large number of samples. The number of samples screened and/or analytes detected simultaneously must be large relative to traditional detection methods.^[^ ^151^ ^]^ Relative to traditional methods, high‐throughput biosensors enable a significant degree of parallelization, making them rapid and cost‐effective.^[^ ^151^, ^152^, ^153^ ^]^ For example, high‐throughput biosensors were developed in response to the SARS‐Cov‐2 pandemic, to enable rapid simultaneous testing of large numbers of samples (i.e., patients).^[^ ^151^, ^152^, ^153^ ^]^ However, relative to traditional single‐sample single‐analyte approaches, high‐throughput biosensors are inherently more complex and can be more difficult to design and implement. Microfluidics represent a possible solution to this problem and could potentially facilitate the design and implementation of high‐throughput biosensing systems.^[^ ^154^, ^155^ ^]^ Microfluidics were developed to replicate laboratory functions on small chips,^[^ ^156^ ^]^ these systems manipulate liquids in micrometer‐scale channels resulting in laminar flow profiles and enhancing heat and mass transport for precise reaction control and shorter reaction times.^[^ ^157^, ^158^ ^]^ Advances in materials and microfabrication techniques have allowed for quick production of chemically inert, thermostable, and low‐toxicity chips.^[^ ^159^, ^160^ ^]^ Microfluidics offer parallelization, multiplexing, precise spatiotemporal control, and reduced sample and energy use,^[^ ^161^ ^]^ and therefore lend themselves well to high‐throughput applications.  Conventional electrochemical sensors typically require large sample and reagent volumes, and bulky instrumentation. Integration of electrochemical sensors with microfluidic chips, and miniaturization of electrochemical equipment could produce electrochemical sensors suitable for high‐throughput applications.^[^ ^162^, ^163^ ^]^ Such high‐throughput devices can produce large, detailed datasets that are difficult to analyze with traditional statistical methods.^[^ ^154^ ^]^ Additionally, while the usability of such devices in POC settings is desirable, for example for screening multiple patients in parallel for a specific disease, their operation and readout may be difficult for a person without the right expertise. Trainable ML models can help process the complex data extracted from these devices and help close the knowledge gap between experts and end‐users.^[^ ^164^ ^]^  In high‐throughput advanced electrochemical biosensors, ML has been used to facilitate drug toxicity screening, to analyze complex signals extracted from microfluidic impedance cytometry, a high‐throughput cell characterization technique,^[^ ^162^ ^]^ and to aid in automating microfluidic device control, with potential applications in high‐throughput systems.

**Figure 7:**
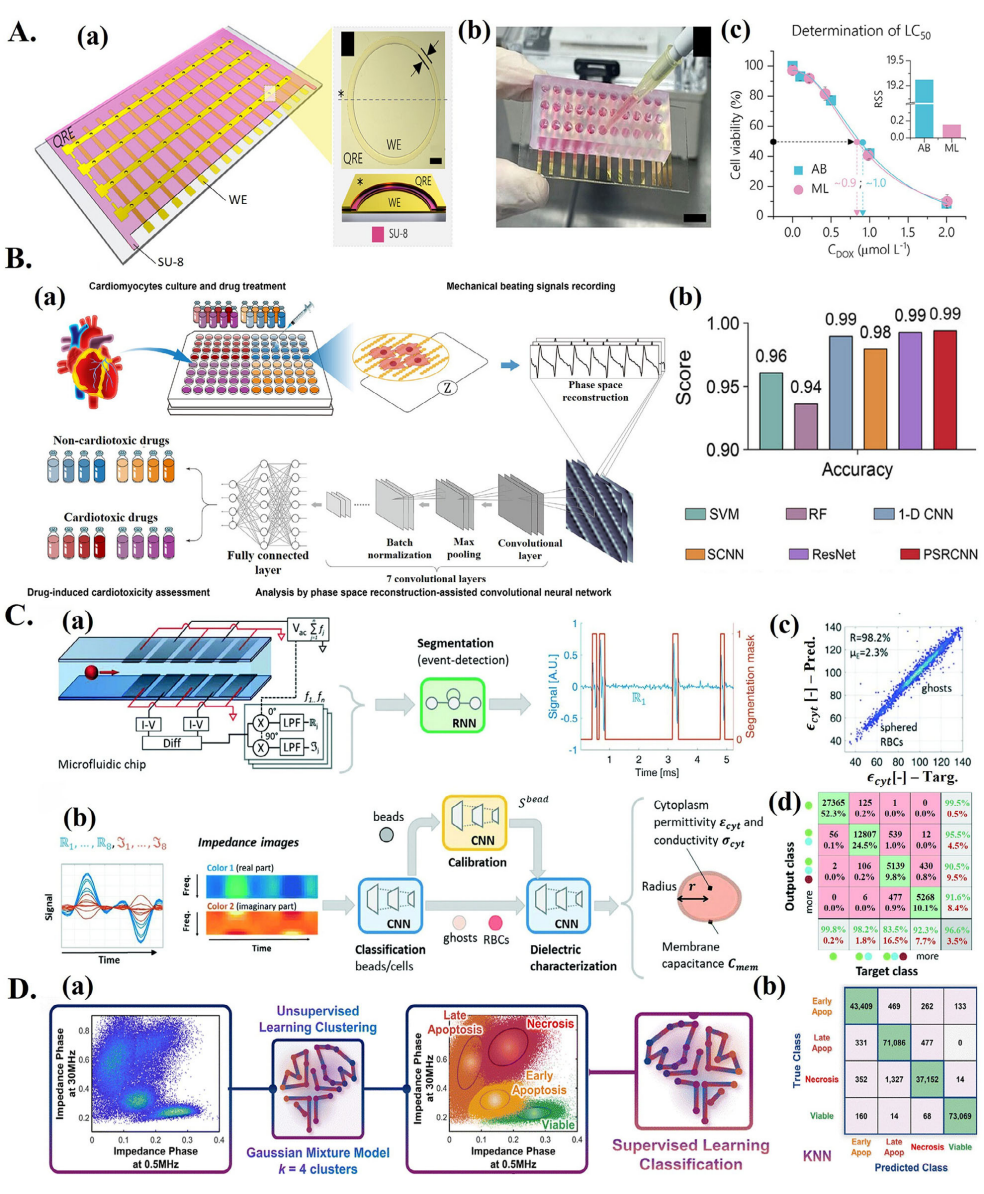
ML‐aided high‐throughput advanced electrochemical biosensors. A) Ultra dense electrochemical high‐throughput chip paired with ML for anti‐cancer drug screening. a) Chip architecture: gold thin‐film WEs on glass substrate, covered with SU‐8 photoresist, which separates WEs from gold thin‐film quasi‐reference electrodes (QREs). b) Multi‐well electrochemical chip filled with media. c) Cell viability as function of doxorubicin concentration, as estimated by (AB) assay, and by ML. Inset showing residual sum of squares (RSS), higher for AB than ML indicating better ML performance. LC_50_ could be obtained by Langmuir fitting of this data. a–c) Reproduced with permission.^[^ ^165^ ^]^ Copyright 2024, American Chemical Society. B) Impedance‐based high‐throughput ML‐aided biosensing platform for drug‐induced cardiotoxicity assessment. a) Overview of platform workflow: culture of cardiomyocytes in sensor plate followed by treatment with several drugs at different concentrations, and high‐throughput impedance recording of cardiomyocytes’ beating signals. Signals are then converted to images using phase space reconstruction and fed to a CNN for analysis and prediction of cardiotoxicity. b) Accuracy of various models for cardiotoxicity prediction (1‐D CNN: 1D CNN, SCNN: scalogram‐assisted CNN). a,b) Reproduced with permission.^[^ ^166^ ^]^ Copyright 2024, John Wiley and Sons. C) Single‐cell phenotyping with ML‐aided impedance cytometer. a) Collection of impedance data from a microfluidic impedance chip, this data is then fed to an RNN trained to identify data stream portions corresponding to flowing particles/cells. b) Signals for each detected particle/cell are reshaped as impedance images and fed into a classification CNN that performs particle classification (beads or cells). Cell impedance images are then processed by a regression CNN that predicts cell size and dielectric properties, whereas bead signals are used to obtain the calibration signal *S^bead^*. c) Predicted versus target cytoplasm permittivity ε_*cyt*_. d) Confusion matrix of coincidence classification into singlet, doublet, triplet, or more (i.e., k‐particle event with k > 3). Normalizations by row (i.e., precision) and by column (i.e., recall) are also reported. a–d) Reproduced with permission.^[^ ^167^ ^]^ Copyright 2022, The Royal Society of Chemistry. D) Classification of pancreatic ductal adenocarcinoma apoptosis stage with ML‐aided impedance cytometer. a) Unsupervised learning clustering using the Gaussian mixture model for identification of subpopulations. Clustered data analyzed by supervised learning. b) Confusion matrix for subpopulation classification using KNN. a,b) Reproduced with permission.^[^ ^168^ ^]^ Copyright 2022, The Royal Society of Chemistry.

Drug toxicity screening is an important application of high‐throughput systems, enabling simultaneous testing of many drug candidates.^[^ ^165^ ^]^ In electrochemical high‐throughput drug toxicity screening systems, ML can be used to facilitate the prediction of drug toxicity from electrochemical signals. For example, Doretto et al.^[^ ^165^ ^]^ proposed an ultra‐dense electrochemical chip paired with ML for high‐throughput anticancer‐drug screening. A meshed electrochemical chip, consisting of dual‐electrode arrayed sensors fabricated by vertical engraving of gold thin‐film electrodes onto glass chips, was used. Gold was chosen due to its high conductivity and good biocompatibility, promoting the adhesion of 2D cell cultures onto the chip. The chip was electrically insulated with a spin‐coated layer of SU‐8 photoresist (**Figure** **7**A,a). Laser‐cut wells allowed for the analysis of 33 120 µL cell‐containing droplets (Figure 7A,b). The chip could also be coupled with microfluidics, by bonding to a 9‐channel PDMS chip, this allowed for testing of up to 9 separate samples on the chip, with each channel covering multiple electrochemical sensors, allowing for technical replicates. The chip was first tested in a multiwell configuration, with exposure of MDA‐MB‐231 breast cancer cells to doxorubicin (DOX). SWV signals were collected for the cells cultured in the different wells. ML was implemented for a more accurate prediction of cell viability (number of live cells): eight SWV currents were used for analysis with SISSO. The *R* ^2^ achieved for the viability prediction task was of 0.994, with accuracies (% recovery) ranging from 98–103%, ML outperformed viability determination via the Alamar Blue (AB) assay (Figure 7A,c and **Table** **5**). The device was then tested in a microfluidic configuration, with HT‐29 colorectal cancer cells treated with DOX. In this configuration, cell detachment from the sensor due to liquid handling, which results in inaccuracy (cells should only be detaching due to death), could be avoided, and reagent consumption could be reduced. In another similar work,^[^ ^166^ ^]^ a high‐throughput platform for cardiotoxicity screening with cardiomyocytes, based on a 96‐well impedance sensor plate was proposed (Figure 7B,a). Interdigitated electrodes (IDEs), onto which cardiomyocytes could attach, were fabricated onto the bottoms of the wells. Cellular impedance was used to record cardiomyocyte beating signals, which were found to vary when exposed to cardiotoxic drugs. For direct analysis of beating signals, deep learning was implemented in the form of a phase space reconstruction‐assisted convolutional neural network (PSRCNN), with phase space reconstruction being utilized to convert 1D beating signals to 2D image representations for analysis with CNN (Figure 7B,a). Cardiomyocytes were treated with various cardiotoxic and non‐cardiotoxic drugs, and the collected signals were analyzed with deep learning models (PSRCNN, 1D‐CNN) and shallow ML models (SVM, RF). The ML models used were trained for various tasks, including binary cardiotoxicity classification, drug type classification, and drug concentration prediction. The deep learning methods (PSRCNN, 1D‐CNN) were found to significantly improve accuracy relative to SVM and RF, for binary cardiotoxicity classification (Figure 7B,b) and other tasks. PSRCNN achieved an *R* ^2^ generally above 0.83 for drug concentration prediction and achieved an accuracy of 82% to 99% for cardiotoxicity prediction of drugs not seen in training data (Table 5). Thus, ML has been paired with electrochemical high‐throughput drug screening systems, for more accurate cell viability prediction, and for direct analysis of electrochemical signals obtained from drug‐treated cells.

**Table 5:** ML‐aided advanced high‐throughput electrochemical biosensors.

Target	Materials	Fluid^a)^	EC method	ML type	Metrics	Year/Refs.
Cell viability	Gold thin‐film electrodes	Cell suspension in culture media	SWV	SISSO	*R* ^2^ (MDA‐MB‐231) = 0.994	2024^[^ ^165^ ^]^
Cardiotoxicity	N.S.	Cell suspension in culture media	Impedimetric	PSRCNN	Acc. (Cardiotoxicity of unseen drugs) = 82% – 99%	2024^[^ ^166^ ^]^
Cell Type	Au/Cr electrodes on glass substrate	Cell suspension in PBS	Impedance cytometry	FCNN	Acc. = 91.5%	2022^[^ ^162^ ^]^
Single‐cell phenotyping	N.S.	Cell suspension in PBS	Impedance cytometry	RNN, CNN	*R* ^2^ (Cell radius, membrane capacitance, cytoplasm permittivity, and conductivity) > 0.98; Acc. (Classification of coincident signals) = 96.6%	2022^[^ ^167^ ^]^
Classification of pancreatic ductal adenocarcinoma apoptosis stage	N.S.	Cell suspension in PBS	Impedance cytometry	KNN	Acc. = 98.4%	2022^[^ ^168^ ^]^
Normal vs cancerous human urothelial cells	Au/Cr electrodes	Cell suspension in PBS	EIS	RF	Acc. = 91.7%	2022^[^ ^169^ ^]^
Cell count, volume, velocity, location in device	Au/Cr electrodes	Cell suspension in PBS	Impedance cytometry	CNN	Qualitative (Comparison of predicted vs true distributions)	2021^[^ ^170^ ^]^

^a)^ Biological fluids are of human origin unless otherwise specified.

Aside from cell response to drug exposure, high‐throughput cell characterization is also of interest, and can be performed with ML‐aided microfluidic impedance cytometry. Feng et al.^[^ ^162^ ^]^ achieved real‐time cell characterization in a microfluidic impedance cytometer using two FCNNs, with computation of cell characteristics requiring 0.3 ms per single‐cell event, indicating potential for high‐throughput applications A two‐layer microfluidic chip was used, with the bottom layer, used for impedance sensing, consisting of coplanar electrodes on a glass substrate, while the top layer consisted of a PDMS constriction microchannel. A regression network computed intrinsic properties (cell radius, cytoplasm conductivity, specific membrane capacitance), from impedance signals. The values for these intrinsic properties were then fed to a classification network for cell type prediction. The proposed neural networks showed good accuracy, with a relative error within 1.3% for cell radius. For intrinsic property estimation, a highly linear relationship between predicted and true values (*R* ^2^ = 0.99) on the test dataset was observed, indicating good prediction performance of the model. When using all three intrinsic parameters for cell type classification, an accuracy of 91.5% was obtained (Table 5).^[^ ^162^ ^]^ A similar study using microfluidic impedance cytometry achieved cell phenotyping using RNN and CNN. The microfluidic impedance chip consisted of a microfluidic channel with multiple micron‐sized electrodes. The RNN segmented the data stream obtained from the chip to detect cell passage events (Figure 7C,a), and the impedance signal corresponding to the detected cell passage was converted into a 2D representation for analysis. The first CNN classified the 2D‐converted impedance signals to differentiate between cell and bead signals. A final regression CNN performed dielectric characterization of the cells (Figure 7C,b), achieving *R* ^2^ above 0.98 for predicting various cell properties (Figure 7C,c, Table 5). Additionally, the proposed device had the capability to resolve coincidence impedance signals by characterizing singlet (single particle), doublet (double particle), and triplet (triple particle) signals, achieving good performance, with a classification accuracy of 96.6% (Figure 7C,d, Table 5). Similarly to the previous work, the prediction times achieved were in fractions of ms, indicating potential for high‐throughput use.^[^ ^167^ ^]^ The ability to conduct rapid and accurate cell analysis holds promise for a wide range of applications, like monitoring immune responses, ultimately contributing to improved healthcare outcomes and more efficient clinical workflows. Impedance cytometry has also been utilized with unsupervised learning techniques for clustering and classification of pancreatic ductal adenocarcinoma cells into early apoptotic, late apoptotic, and necrotic subpopulations under drug treatment. Hypotonic treatment of cells was used to induce varying degrees of apoptosis. Unsupervised learning first identified clusters corresponding to viable, early apoptotic, late apoptotic, and necrotic cells (Figure 7D,a). Subsequently, supervised learning was used to train a KNN classifier to categorize gemcitabine‐treated cells based on impedance data (Figure 7D,a), achieving an accuracy of 98.4% (Figure 7D,b, Table 5).^[^ ^168^ ^]^ This approach led to novel findings related to the tumor microenvironment and methods to prevent the creation of an immunosuppressive environment. In another study, researchers developed a microfluidic impedance cytometry device that employed EIS combined with ML to differentiate between healthy and cancerous human urothelial cells. Statistically significant impedance differences were found between normal and cancerous cells. Various machine learning models were trained and tested on impedance spectra, discovering that a RF model delivered the best performance, achieving an accuracy of 91.7% (Table 5). This approach not only highlighted the potential of integrating EIS with ML for precise cell differentiation but also opened new avenues for early cancer detection and improved diagnostic methods.^[^ ^169^ ^]^ The short read times that can be obtained with microfluidic impedance cytometry enable high‐throughput cell characterization, and by coupling with ML, information such as cell type, disease classification, or cell apoptosis stage can be extracted from subtle patterns present in impedance data.

Aside from analysis of impedance cytometry signals, ML can also aid in microfluidic device control, which can be highly beneficial in high‐throughput applications, where the large amount of parallelized tests make manual operation difficult and/or impossible.^[^ ^154^ ^]^ Automated control can be achieved by ML models that have learned to detect events and activate dynamic components in response.^[^ ^164^ ^]^ In an innovative development, researchers created an adaptive microfluidic system designed to manage specimen heterogeneity and respond to internal and external perturbations. The system could adapt through distributed electrical sensors able to detect changes in current as cells moved over electrodes. A closed‐loop feedback control system was then employed to modulate chip parameters based on this sensor data. The current data was processed in real‐time, with CNN algorithms interpreting the information to determine cell count, volume, velocity, and location within the device. A feedback controller used this analysis to update a programmable pressure pump, ensuring a desired cell flow speed. The system showed good predictive ability for the various cell parameters and quickly converged to both static and dynamic flow targets, demonstrating its capability for precise and adaptive cell management.^[^ ^170^ ^]^ ML thus shows potential for automating control of microfluidic electrochemical sensors, a necessity if such devices are to be used successfully in high‐throughput applications.

Whether it be for the assessment of cell viability in response to drug treatment, or for cell characterization through microfluidic impedance cytometry, ML has enabled the extraction of relevant quantitative or qualitative information from the complex signals generated by high‐throughput electrochemical biosensors. The use of deep learning models to process these signals has been especially prominent. The use of ML for automation of flow control in microfluidic devices is also highly interesting, and may enable automated, microfluidics‐based, high‐throughput systems. To the author's knowledge, applications of ML in high‐throughput electrochemical biosensors have remained limited. Increasing miniaturization of electrochemical sensors,^[^ ^149^, ^150^ ^]^ and the transition of current lab‐based electrochemical sensors to portable *lab‐on‐chip* setups^[^ ^162^, ^163^ ^]^ are likely to benefit from ML integration to help maintain sensor reliability despite miniaturization, and this will likely lead to more advances in high‐throughput electrochemical biosensors. The need for miniaturization has also been felt in continuous monitoring electrochemical biosensors, which face similar challenges as high‐throughput biosensors, and which could also strongly benefit from ML integration.

### 3.6 Continuous Monitoring Biosensors

Medical diagnostics rely heavily on laboratory testing, which often requires invasive blood sampling, dedicated personnel, and expensive equipment.^[^ ^171^, ^172^ ^]^ This forces a more reactive approach to healthcare, where testing is often carried out in reaction to the appearance of severe symptoms. This, in turn, makes early disease detection and treatment challenging if not impossible.^[^ ^173^ ^]^ The ability to continuously monitor key disease biomarkers would enable a more proactive diagnostic and treatment approach by allowing early detection of abnormal biomarker values.^[^ ^173^ ^]^ This would also ease the management of chronic illnesses, such as diabetes, gout, and Parkinson's. Interest in biosensors capable of continuous monitoring has therefore been increasing, and electrochemical continuous monitoring biosensors are of particular interest due to their ease‐of‐use and quick response. Wearable biosensors, worn unobtrusively on the body with the aim of detecting key biomarkers in surface‐accessible bodily fluids (sweat, tears, and interstitial fluid),^[^ ^174^, ^175^ ^]^ greatly facilitate continuous monitoring. These biosensors can further be paired with miniaturized wireless telemetry to enable remote monitoring and pairing with smartphones and smartwatches.^[^ ^174^, ^176^ ^]^ Continuous monitoring biosensors inherently produce large quantities of data, and early signs of disease can often be present as subtle changes that must be found within this large data.^[^ ^36^, ^177^ ^]^ ML is well suited for analysis of the highly dimensional, large, and often noisy datasets produced by these sensors, and is capable of detecting the subtleties indicative of early‐stage disease.^[^ ^178^, ^179^, ^180^ ^]^ As continuous monitoring biosensors are often wearable, variations in ambient conditions (e.g., temperature, humidity, and pH) can often introduce noise and non‐linearities into the electrochemical signal,^[^ ^31^ ^]^ ML has the capacity to deal with these issues.

**Figure 8:**
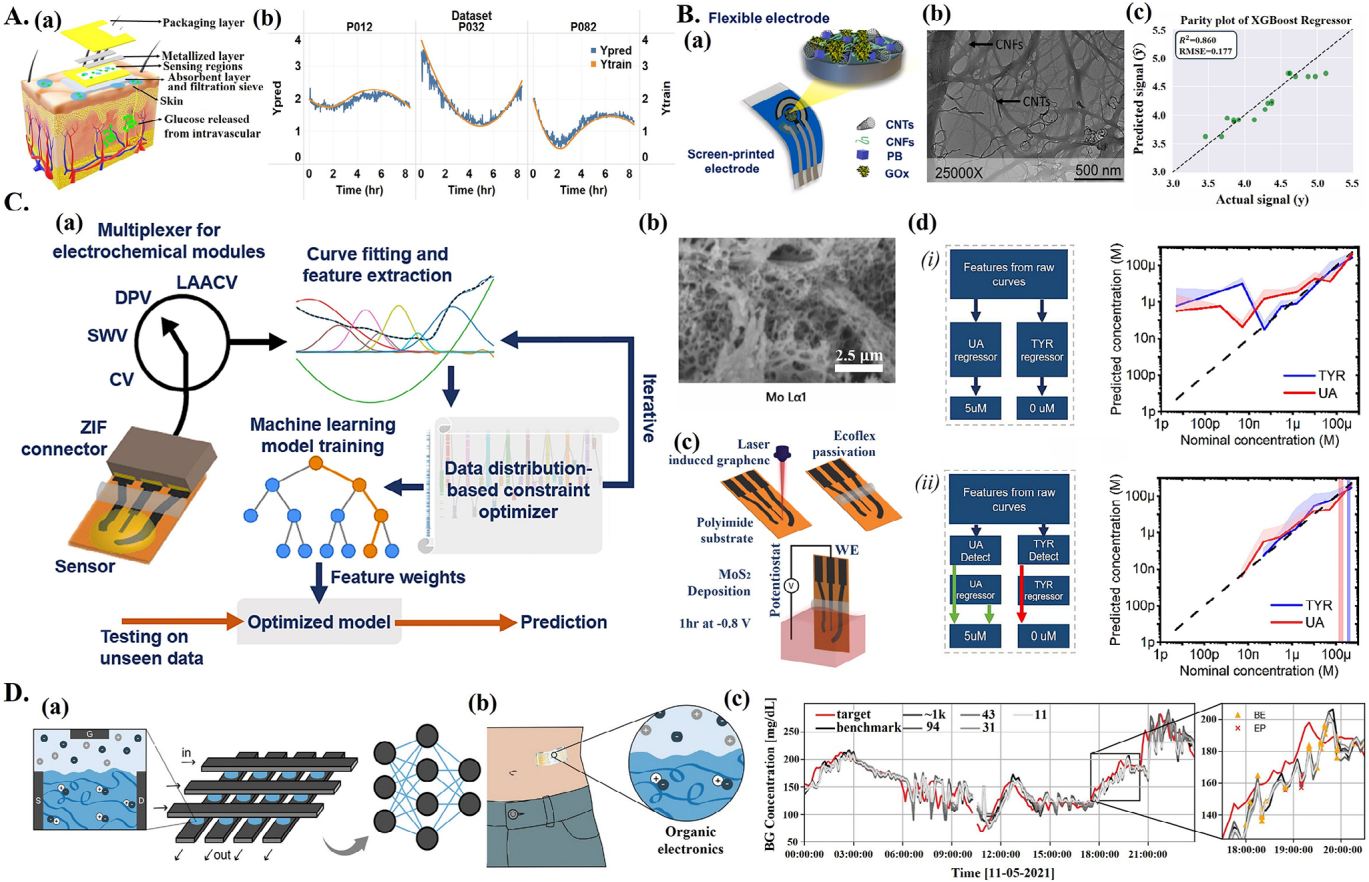
ML‐aided advanced electrochemical continuous monitoring biosensors. A) ML‐aided wearable EIS sensor for glucose continuous monitoring. a) Sensor assembly. b) Comparison of predicted glucose values to reference glucose values (Ytrain) for three human subjects. a,b) Reproduced with permission.^[^ ^181^ ^]^ Copyright 2022, Springer Nature. B) Body‐strap advanced electrochemical sensor for real‐time sweat glucose detection with ML‐aided sensor optimization. a) Sensor electrode modified with CNT – CNF, PB, and GOx. b) TEM image of CNTs – CNFs composite. c) XGBoost regressor performance for signal prediction. a–c) Reproduced with permission.^[^ ^183^ ^]^ Copyright 2024, Elsevier. C) ML‐aided multi‐modal electrochemical wearable sensor for UA and TYR detection. a) Overview of device workflow. b) SEM image of eMoS_x –_ LIG material. c) Overview of device fabrication. d) i) Structure (left) and performance (right) of one‐step regressor. ii) Structure (left) and performance (right) of two‐step classifier‐regressor, with physiological TYR and UA ranges shown in vertical colored bars. a–d) Reproduced with permission.^[^ ^33^ ^]^ Copyright 2022, Elsevier. D) ENODe integrated with ML for blood glucose detection. a) From left to right: ENODe schematic, showing source (S), gate (G), and drain (D) terminals, crossbar array of ENODe devices, hardware‐based neural network with individual ENODes as network weights. b) Illustration of wearable organic electronics. c) Blood glucose prediction of ENODe‐based models with various sizes (number of parameters), as compared to benchmark (black) and target (red) values. a–c) Reproduced with permission.^[^ ^19^ ^]^ Copyright 2024, John Wiley and Sons.

Multi‐modal ML has been applied to continuous monitoring electrochemical biosensors for improved performance through the integration of operating condition data with electrochemical data. For example, Sardesai et al. proposed an ML‐aided wearable electrochemical sensor for continuous sweat‐based monitoring of glucose and cortisol. The device made use of a semiconductor electrode layer of 100–200 nm thick zinc oxide film. Impedance data, relative humidity, and skin temperature data were fed to an ensemble regression model for glucose and cortisol concentration prediction, achieving an *R* ^2^of 0.96 (**Table** **6**). In another work by the same group,^[^ ^181^ ^]^ a proof‐of‐concept wearable skin biosensor for continuous glucose monitoring based on EIS readings from eccrine sweat was proposed (**Figure** **8**A,a). Impedance readings were combined with independently measured skin temperature and relative humidity values and fed to various ML algorithms for the prediction of sweat glucose concentration. To improve generalization, white noise was introduced at a signal‐to‐noise ratio of 10 dB. A DT model was used for sweat glucose concentration prediction, achieving an *R* ^2^ of 0.93 and RMSE of 0.1 mg dL^−1^ (Table 6). The proposed model could closely track real patient glucose concentrations over time (Figure 8A,b). Finally, Bao et al.^[^ ^31^ ^]^ proposed an advanced ML‐aided wearable device for tyrosine (TYR) detection, with potential for use in continuous monitoring. The device consisted of carbon‐black and graphene‐oxide (CB‐GO) paper‐based electrodes connected to a small flexible electrochemical analysis device. While a calibration curve based on DPV data provided reliable TYR concentration prediction, accuracy suffered as temperature and pH conditions varied. ML was introduced to correct for these variations, with a ridge regression model achieving an *R* ^2^ of 0.9828 and RMSE of 5.4170 µM for TYR concentration prediction in artificial urine (Table 6). ML can therefore integrate operating condition data to compensate for variable operating conditions in continuous monitoring sensors. Extended on‐body testing of the proposed sensors will be necessary to verify that these ML‐aided sensors can maintain good predictive performance over time.

**Table 6:** Machine‐learning‐aided advanced electrochemical biosensors for continuous monitoring.

Target	Materials	Fluid^a)^	EC method	ML type	Metrics	Year/Refs.
TYR	CB – GO conjugate polymer WE	Artificial urine	DPV	Ridge regression	*R* ^2^ = 0.9828; RMSE = 5.4170 µm	2023^[^ ^31^ ^]^
Glucose	N.S.	Sweat	EIS	DT	*R* ^2^ = 0.93; RMSE = 0.1 mg dL^−1^	2022^[^ ^181^ ^]^
Glucose, Cortisol	Zinc oxide film WE	Sweat	Non‐faradaic EIS	Ensemble regression	*R* ^2^ = 0.96; RMSE = 0.4 ng mL^−1^	2023^[^ ^193^ ^]^
Core body temperature	Ag/AgCl ink electrodes, covered with PVC‐based ion selective membrane	Sweat	Potentiometry	RF	RMSE = 0.02 ^○^C	2023^[^ ^182^ ^]^
Current response signal	WE modified with PB/CNT – CNF/Chitosan	H_2_O_2_ solution	Amperometry	XGBoost	*R* ^2^ = 0.860; RMSE = 0.177 µA	2024^[^ ^183^ ^]^
Glucose	Organic polymer‐based electronics	Blood	ENODe	FCNN	31‐parameter ENODe‐based ANN: Accurate predictions (CG‐EGA)^[^ ^191^ ^]^ = 84.85%; RMSE = 23.19–24.02 mg dL^−1^	2024^[^ ^19^ ^]^
TYR, UA	eMoSx – LIG WE	Artificial saliva, artificial sweat	DPV, SWV, LAACV	DT	LOD = 100 nm; LOD (UA) = 10 nm	2022^[^ ^33^ ^]^

^a)^ Biological fluids are of human origin unless otherwise specified.

In some cases, electrochemical data may not be sufficient for inference of the target variable, and additional information may need to be extracted from other data modalities. Wang et al.^[^ ^182^ ^]^ proposed a wearable electrochemical sensor for continuous monitoring of sweat biomarkers, integrating sweat NA^+^ and K^+^ concentration data with physiological markers for core body temperature prediction during exercise. Sweat NA^+^ and K^+^ sensors were integrated with a sweat‐channeling microfluidic component and paired to a portable readout system for live data acquisition. The potentiometric ion sensors were composed of printed Ag/AgCl ink. A PVC‐based ion selective membrane was drop‐coated onto individual sensing electrodes to achieve selective ion detection. The device was tested on‐body for the collection of NA^+^ and K^+^ concentration time series data. This data was combined with physiological markers (i.e., heart rate, regional sweat rate, power measured by ergocycle) and used to train various ML models for core body temperature prediction, after applying PCA for dimensionality reduction. Out of all models tested, RF regression gave the best results, with an RMSE of 0.02^○^C for core body temperature prediction (Table 6), an improvement over previous similar work. Interestingly, this result was achieved while only using Na^+^ and K^+^ concentrations as input features, indicating that multi‐modal learning was not necessary in this case, and that electrochemical data were sufficiently descriptive. Still, this work demonstrates the possibility of combining electrochemical data with physiological data, which may prove useful in other applications.

Multi‐modal learning has also been used to combine data from different electrochemical sensing modalities in wearable sensors. Kammarchedu et al.^[^ ^33^ ^]^ developed an advanced wearable electrochemical biosensor for ML‐aided detection of UA and TYR in sweat and saliva, with potential for use in continuous monitoring (Figure 8C,a). The wearable sensor was based on a working electrode composed of electrodeposited molybdenum polysulfide (eMoSx) on LIG (Figure 8C,b,c). As opposed to bare LIG, the electrodeposition of molybdenum polysulfide increases the electrochemically active surface area and leads to improved sensitivity to TYR and UA. DPV, SWV, and large amplitude AC voltammetry (LAACV) data were collected for training and testing of various ML models, with DT regression showing the best performance. Direct TYR and UA concentration prediction with two separate models were found to perform poorly (Figure 8C,d(i)). Instead, a two‐step architecture with initial binary detection (classification) of the analytes, followed by concentration prediction (regression), was implemented (Figure 8C,d(ii)). This allowed for LOD of 100 and 10 nm for TYR and UA, respectively (Table 6), two orders of magnitude lower than analysis methods relying on a single feature extracted from a single type of data. While the integration of multiple electrochemical data modalities allowed for a lower LOD, the data collection hardware (potentiostats, potentiostat multiplexer) remained quite bulky, with further miniaturization being required for implementation of this approach in a device suitable for continuous monitoring.

Aside from the analysis of biosensor data, there is potential for applying ML to aid in biosensor design.^[^ ^2^ ^]^ Promphet et al.^[^ ^183^ ^]^ proposed a body‐strap sensor for real‐time sweat glucose detection, with an integrated portable potentiostat connecting via Bluetooth to a smartphone. The sensing strip working electrode was modified with a carbon nanotube – cellulose nanofiber (CNF) composite followed by Prussian blue electrodeposition (Figure 8B,a,b). Modification with CNT resulted in increased specific surface area of the electrode, which allowed for a higher enzyme loading and improved electron transfer between the electrode surface and redox species. Combination of CNTs with CNFs allowed for improved dispersion of CNTs through electrostatic stabilization and led to enhanced enzyme loading and improved sweat absorption. Finally, a coating of chitosan on the top layer of the electrode strip helped preserve the activity of the GOx enzyme used. To optimize the sensor design, an XGBoost model was trained to predict the current response signal of 1 mm H_2_O_2_, as a function of CNT and CNF concentrations ([CNF], [CNT]) and of the number of Prussian blue deposition cycles. Pearson's correlation coefficients between the different features were first computed, showing a negative correlation between the signal and [CNF], while [CNT] and the number of cycles showed weaker positive correlations. To interpret model predictions, the SHappley Additive exPlanations (SHAP) framework was used. SHAP is based on game theory and, for a given prediction, assigns each input feature an importance score, allowing for the identification of the most important features, in the “eyes” of the model, for that specific prediction.^[^ ^184^ ^]^ The SHAP analysis indicated a higher overall importance of [CNF] relative to the other features, shown by higher SHAP attribution values. The trained model achieved an *R* ^2^ of 0.860 and RMSE of 0.177 (Figure 8B,c, Table 6) for current response signal prediction. This work represented a rare example, in the field of ML‐aided electrochemical biosensors, of the use of an explanatory framework such as SHAP to clarify black box model predictions. ML implementation thus shows potential for understanding the variables at play in achieving optimal electrochemical sensor design, and for aiding in optimization of electrochemical sensors.^[^ ^2^, ^3^, ^31^ ^]^

A key consideration in the design of continuous monitoring sensors is size, as lightweight, compact, and unobstructive hardware can facilitate continuous monitoring.^[^ ^173^, ^185^ ^]^ However, this hardware must also remain capable of supporting complex ML models.^[^ ^19^ ^]^ This becomes especially critical as larger datasets are acquired through continuous monitoring, requiring more complex models for data analysis.^[^ ^186^ ^]^ Organic polymer‐based electronics have shown potential as novel materials for use in continuous monitoring applications; they are flexible, stretchable, biocompatible and operate at low voltages,^[^ ^187^, ^188^, ^189^ ^]^ facilitating wearability, and by extension, continuous monitoring. Kurt et al.^[^ ^19^ ^]^ proposed a hardware ANN, implemented within an electrochemical neuromorphic organic device (ENODe) for the prediction of blood glucose levels. The ENODe was composed of source (S), drain (D), and gate (C) terminals with an electrolyte enabling ionic connections to the gate. Multiple ENODe devices were arranged into crossbar arrays, with each ENODe representing a weight in the hardware neural network (Figure 8D,a), ANN implementation with this architecture could enable on‐body computation (Figure 8D,b). Complexity reduction of the ANN was necessary for ENODe‐based hardware implementation, including bounding and discretization of the possible number of states for the neural network weights. The blood glucose prediction performance was tested on data from individuals in the OhioT1DM dataset.^[^ ^190^ ^]^ As the model size was decreased and the parameter space was reduced, RMSE generally increased, showing limitations for model down‐sizing. Blood glucose predictions over time for models with varied sizes closely followed the benchmark while capturing the trend in target values (Figure 8D,c). As per the CG‐EGA^[^ ^191^ ^]^ performance metric, the 31‐parameter ENODe‐based ANN achieved 84.85% accurate predictions on Cohort 2018 of the OhioT1DM dataset (Table 6), compared to 80.4% for the benchmark. Integration of ML with flexible organic electronics may help achieve more flexible and comfortable wearable electrochemical sensors while maintaining the required processing power, these sensors will then be more suitable for continuous monitoring applications.

As the need for a more proactive approach to healthcare demands more frequent tracking of key disease biomarkers, electrochemical diagnostics are shifting from bulky laboratory‐based hardware to more portable, often on‐body, hardware. This new mode of operation is more prone to variations in ambient environmental conditions, which decrease the accuracy of traditional calibration curve approaches for signal analysis.^[^ ^58^, ^192^ ^]^ ML has shown the ability to manage these variations by integrating operating condition data through multi‐modal learning. ML can also aid in sensor design, can be integrated with organic electronics, and can enable multi‐modal data analysis. In order to quantify gains in ML model performance derived from the introduction of additional data modalities, single‐modal baselines should first be established, as seen in other work.^[^ ^139^, ^146^ ^]^ Moving forward, ML will likely be key in providing automated analysis of advanced electrochemical continuous monitoring sensor data, but the size of ML models will need to be tailored to hardware limitations of wearable sensors. The growing integration of hybrid sensing modalities, combining biophysical and biochemical sensors, significantly increases the computational demands of ML models. To address this, technologies need to prioritize efficient on‐board data processing within compact, space‐constrained devices. Alternatively, high‐fidelity data transmission could be leveraged to enable ML model processing in the cloud or on an external high‐performing computing device. The introduction of advanced materials will also likely be key in bringing forth the next generation of continuous monitoring sensors. Current commercially available sensors are often restricted to monitoring of highly concentrated analytes (e.g., glucose).^[^ ^171^ ^]^ Introduction of advanced/nanomaterials can increase sensor sensitivity and enable the monitoring of more dilute analytes.^[^ ^8^ ^]^ Advances in organic electronics may also facilitate continuous monitoring by improving wearability.^[^ ^7^, ^19^ ^]^

## 4 Conclusion, Challenges, and Outlook

Machine learning, paired with advanced materials, has shown promise for enhancing the performance of electrochemical biosensors. In biocatalytic electrochemical sensors, advanced materials, such as multiwalled carbon nanotubes, gold nanoparticles, clay particles, and MoS_2_ have been used to improve sensor sensitivity and specificity, to enable the detection of multiple analytes without requiring multiple biorecognition elements, and to replace enzymes altogether.^[^ ^76^, ^77^, ^78^ ^]^ These sensors have benefited from multi‐modal ML to maintain sensor performance in the presence of decreasing enzyme activity and variability in operating conditions.^[^ ^22^, ^62^ ^]^ In affinity sensors, variability can also be introduced due to weak affinity of the biorecognition element for the analyte, here ML has been used to analyze lower‐quality data resulting from such weak interactions.^[^ ^100^ ^]^ Moreover, ML has also been applied for direct analysis of impedance spectra obtained from EIS,^[^ ^101^ ^]^ the transduction method of choice for affinity sensors. ML‐aided affinity sensors have made use of advanced materials such as multiwalled carbon nanotubes, gold nanoparticles, laser‐scribed graphene, and carbon nanotube thin films, in the case of the latter, the variability inherent to the material could be reduced by introducing ML, indicating the relevance of merging advanced electrochemical sensors with ML.^[^ ^101^, ^102^, ^106^ ^]^ While biorecognition elements help maintain sensor specificity, detection is limited to a single analyte per recognition element, and the biomolecule synthesis process is complex and expensive;^[^ ^6^, ^110^ ^]^ ML has been used to enable bioreceptor‐free electrochemical sensors by resolving analytes with overlapping signals on unmodified electrodes and analyzing “fingerprints” produced by electrochemical sensor arrays.^[^ ^21^, ^30^, ^115^, ^122^ ^]^ These bioreceptor‐free sensors have included materials such as graphene oxide, silver nanowires, and vapor‐grown carbon fiber microelectrodes to improve the electrochemical signal and improve sensitivity.^[^ ^113^, ^114^, ^115^ ^]^ ECL‐based sensors have similarly used materials such as laser‐scribed graphene, graphene oxide, and gold nanoparticles, for enhanced sensitivity and stability.^[^ ^140^, ^146^ ^]^ These sensors have applied multi‐modal ML to combine ECL data with other electrochemical sensing modalities for enhanced sensor performance.^[^ ^145^, ^146^ ^]^ In high‐throughput electrochemical biosensors, ML, and especially deep learning, have enabled the extraction of quantitative or qualitative information, such as drug cardiotoxicity, cell viability, and cell type, from large sets of complex electrochemical signals generated by high‐throughput sensors.^[^ ^162^, ^165^, ^166^, ^167^, ^170^ ^]^ Signal complexity and dataset size can also be problematic with continuous monitoring electrochemical sensors, which have benefited from ML for improved performance through the integration of electrochemical data with operating condition data (e.g., temperature, pH),^[^ ^31^, ^181^ ^]^ and through the combination of multiple electrochemical data modalities.^[^ ^33^ ^]^ Finally, aside from analysis of sensor response, ML has also been useful in electrochemical biosensor design, by predicting the sensor response as a function of different sensor design variables,^[^ ^183^ ^]^ and by helping identify optimal strategies for biorecognition element immobilization.^[^ ^194^ ^]^ Therefore, ML has demonstrated its potential when paired with various types of advanced electrochemical biosensors.

Despite its potential to enhance data analysis and detection accuracy, the integration of ML with electrochemical biosensors still presents several challenges. One key challenge lies in the variability and noise inherent in electrochemical signals, which can complicate the training of even robust ML models. The quality of the data from biosensors often depends on various factors such as temperature, pH, and electrode surface conditions, leading to inconsistencies that can impact model performance. Additionally, collecting large, high‐quality datasets, necessary for training complex ML models, can be time‐consuming and resource‐intensive. To demonstrate the generalizability of ML‐aided electrochemical sensors to clinical samples, datasets must inevitably be reflective of the diversity and variability of patient samples. To obtain such data in sufficient quantities is not trivial, as access to data collected from patient samples is often limited. The application of ML‐aided electrochemical biosensors in clinical settings also introduces a number of ethical and regulatory concerns. Access to patient data, required for ML model training, comes with concerns related to privacy and informed consent; data sharing must be carefully managed so as not to put patient privacy at risk.^[^ ^195^, ^196^ ^]^ If the training data is not truly representative of the populations targeted by the diagnostic device, the model may learn biases, whereby specific populations could be misdiagnosed at a higher rate than others due to under‐representation in the training data.^[^ ^195^, ^196^, ^197^ ^]^ Accountability is also a concern: if an ML‐aided diagnostic device makes an incorrect diagnosis that has harmful consequences, who is to be held accountable?^[^ ^195^, ^196^, ^197^ ^]^ Accountability, and analysis of misdiagnoses, are made more difficult by the “black‐box” nature of many ML models, which can also make it difficult to detect faults in the algorithm, potentially leading to misdiagnoses and subsequent harm.^[^ ^196^ ^]^ Application of such ML‐aided sensors in clinical settings will require increased trust of clinicians in model predictions. Ethics‐based frameworks for the design of ML tools, as well as increased regulation of these tools, are needed to mitigate these ethical and regulatory concerns.^[^ ^195^, ^196^, ^197^ ^]^ More studies using explainable tools to decipher the black box or a shift towards designing electrochemistry biosensors studies incorporating transparent ML analysis is needed to boost translation. Another challenge is the need for domain‐specific expertise to develop models that accurately interpret electrochemical data, as ML techniques often require careful tuning and validation to ensure reliable outputs. Without care, data leakage can occur,^[^ ^198^ ^]^ whereby model evaluation data is not truly unseen, resulting in overly optimistic estimates of model performance during evaluation. Splitting of technical replicates from the same patient sample between training and test sets is an example of data leakage; this leads to a lack of reproducibility of model results and produces models that fail to generalize.^[^ ^43^ ^]^ Another challenge with electrochemical sensors, is miniaturization for improved portability, which is necessary for POC applications. Pairing microfluidics with electrochemical sensors can drive further miniaturization, and ML has been useful for the analysis of the complex signals produced by such devices.^[^ ^168^, ^169^ ^]^ Miniaturization is particularly important in continuous monitoring sensors, but it can be challenging to achieve while maintaining sensor performance, especially as such sensors can be subject to highly variable operating conditions due to their on‐body operation. Furthermore, real‐time deployment of ML models on low‐power hardware, often required for portable electrochemical biosensors, adds an additional layer of complexity. Addressing these challenges requires a multidisciplinary approach that combines expertise in chemistry, data science, and engineering to unlock the full potential of ML‐enhanced advanced electrochemical biosensors.

Moving forward, the integration of advanced materials and ML with electrochemical biosensors will likely become increasingly important, as advanced materials will help improve sensor sensitivity while ML will help improve the robustness and specificity of these sensors. Multidisciplinary collaborative efforts will help advance ML‐aided electrochemical biosensors; further collaboration with clinicians will allow for the testing of devices in clinical settings and will help facilitate access to patient samples. Larger datasets which include patient sample data could enable predictive models that are more robust to the variability and noise inherent to electrochemical signals and signals extracted from biological samples. In cases where obtaining such datasets is unfeasible, data augmentation and data generation techniques, which have been applied to spectral data,^[^ ^199^, ^200^ ^]^ could artificially increase the size of existing datasets. Transfer learning approaches, which pre‐train a model on a large, general, dataset and then fine‐tune the same model on a smaller task‐specific dataset,^[^ ^201^ ^]^ may also help overcome data scarcity; large datasets collected from spiked synthetic samples could be used for pre‐training, with fine‐tuning on limited patient data. Multi‐modal learning is another possible solution for data scarcity and high variability in electrochemical biosensor signals and will likely be increasingly used. As ML‐aided electrochemical biosensors advance, pre‐processing approaches will likely evolve as well. While current approaches often rely on feeding features extracted manually from electrochemical data to conventional ML algorithms (e.g., RF, SVM), ML models can also be trained with features extracted from raw electrochemical data by dimensionality reduction methods, such as PCA. Deep learning approaches, however, can integrate feature extraction within the predictive model, automatically learning relevant data representations from raw data.^[^ ^202^ ^]^ This eliminates the need for domain expertise in selecting relevant features, simplifying computational pipelines, and providing richer data to the model. ML‐aided electrochemical biosensors may therefore benefit from deep learning approaches, though sufficient data will be needed to train these models. Caution must still be exercised in selecting the appropriate model: for applications where data is scarce, a shallow ML model may still be preferable to a data‐hungry deep learning model. Nonetheless, the successful application of any predictive model in a clinical setting will require clinicians’ trust; frameworks such as SHAP,^[^ ^184^ ^]^ LIME,^[^ ^203^ ^]^ and DeepLIFT,^[^ ^204^ ^]^ are explainable tools that aim to explain ML model predictions, which will help achieve this. As electrochemical biosensors move away from laboratory‐based applications,^[^ ^22^ ^]^ maintaining sensor performance becomes difficult in the face of decreasing control over sensor operating conditions and miniaturization. As ML expertise among researchers increases, and access to the necessary computational resources improves, ML will likely become increasingly important in overcoming such challenges in electrochemical biosensors.

## Conflict of Interest

S.S.M. is affiliated with Beeta Biomed, a company that helps with commercialization of the patented technologies in the area of diagnostic devices and biosensors. The other authors declare no conflict of interest.
